# Global Phosphoproteomic Analysis Reveals the Defense and Response Mechanisms of *Jatropha Curcas* Seedling under Chilling Stress

**DOI:** 10.3390/ijms20010208

**Published:** 2019-01-08

**Authors:** Hui Liu, Fen-Fen Wang, Xian-Jun Peng, Jian-Hui Huang, Shi-Hua Shen

**Affiliations:** Key Laboratory of Plant Resources, Institute of Botany, Chinese Academy of Sciences, Beijing 100093, China; huiliu@ibcas.ac.cn (H.L.); wangfenfen@ibcas.ac.cn (F.-F.W.); pengxianjun@ibcas.ac.cn (X.-J.P.); jhhuang@ibcas.ac.cn (J.-H.H.)

**Keywords:** *Jatropha curcas*, phosphoproteomics, seedling, chilling stress, regulated mechanism

## Abstract

As a promising energy plant for biodiesel, *Jatropha curcas* is a tropical and subtropical shrub and its growth is affected by one of major abiotic stress, chilling. Therefore, we adopt the phosphoproteomic analysis, physiological measurement and ultrastructure observation to illustrate the responsive mechanism of *J. curcas* seedling under chilling (4 °C) stress. After chilling for 6 h, 308 significantly changed phosphoproteins were detected. Prolonged the chilling treatment for 24 h, obvious physiological injury can be observed and a total of 332 phosphoproteins were examined to be significantly changed. After recovery (28 °C) for 24 h, 291 phosphoproteins were varied at the phosphorylation level. GO analysis showed that significantly changed phosphoproteins were mainly responsible for cellular protein modification process, transport, cellular component organization and signal transduction at the chilling and recovery periods. On the basis of protein-protein interaction network analysis, phosphorylation of several protein kinases, such as SnRK2, MEKK1, EDR1, CDPK, EIN2, EIN4, PI4K and 14-3-3 were possibly responsible for cross-talk between ABA, Ca^2+^, ethylene and phosphoinositide mediated signaling pathways. We also highlighted the phosphorylation of HOS1, APX and PIP2 might be associated with response to chilling stress in *J. curcas* seedling. These results will be valuable for further study from the molecular breeding perspective.

## 1. Introduction

As one of the most critical limiting factors, low temperature affects the plant growth and development broadly as well as yield, quality, postharvest life and geographic distribution [1]. Chilling tolerance is the ability of a plant to tolerate low temperature (0–15 °C) without injury or damage [2]. Although it is possible to enhance the physical and physiochemical tolerance according to cold acclimation [3], plant species origin from tropical and subtropical areas, such as *Oryza sativa*, *Zea mays* and *Lycopersicon esculentum*, are sensitive to chilling stress and easily damaged by chilling temperature [4]. The cellular structure and physiological characterization of tropical plants were both changed in response to chilling stress, especially the photosynthetic organelle-chloroplast [5,6,7]. Compared to PSI, PSII was likely more sensitive to chilling temperature under moderate light in tropical trees. In order to protect PSI at such stress situation, the PSII photoinhibition appeared and PSII reaction centers was closed [6]. Subsequently, the recovery of PSII from low temperature depended on its ability to maintain PsaA, Cyt b6/f and D1 protein at photoinhibitory conditions [8]. Low temperature stress induced the changes of a variety of protein kinases and transcription factors in plants [5,9,10]. It was reported that increases in the cytosolic transient calcium flux played a vital role in an early step of cold stress signaling [11,12,13]. ABA was responsible for the stomatal closure as well as low temperature stress responses [14] and SnRK2s were active protein kinases that participated in the regulation of ABA [15]. ABI5 and TRAB1 both were bZIP transcriptional factors and had been demonstrated to have the ability to mediated ABA signals [16,17,18,19]. HOS1 was another important negative regulator of cold stress signaling in plant cells [20], as well as acted as an E3 ligase to be required for the ubiquitination of ICE1 [21] and appeared to act upstream of CBF transcription.

As the major and reversible post-translational modification, protein phosphorylation was crucial for providing the basis for complex signaling networks and the regulation of diverse cellular functions in plants [22]. Phosphorylation mainly regulated kinases and phosphatases in a dynamic process, including signal transduction, homeostasis, protein degradation, metabolism and stress responses [23]. With the rapid development of proteomic technology, determination of phosphorylation sites mainly employed LC-MS/MS without gel-based [24]. Additionally, with the development of functional genomes and mass spectrum tools, it was practicable to identify and quantify the protein phosphorylation on a large scale [25]. Therefore, plant protein phosphorylation events played important roles in designing strategies to prevent crops from biotic and abiotic stresses, in particular, quantitative phosphoproteomics should be taken into consideration when illustrate the stress-induced related signal pathways [26]. It has been widely used for illustrating phosphorylation networks in *Arabidopsis thaliana*, *Brachypodium distachyon*, *Triticum aestivum*, *Broussonetia papyrifera* after hormonal and dehydration treatment, salt and chilling stress [27,28,29,30]. 

*Jatropha curcas* L., which originated from the tropics or subtropics, is a woody oil plant that belongs to the Euphorbiaceae family [31]. Due to the abundant oil content in its seeds (as high as 40%), *J. curcas* has been considered as the ideal material for biodiesel in the world [32]. Taking the advantage of the high-throughput technologies, a great body of information on *J. curcas* has been achieved with the application of genomic [33,34], transcriptomic [35,36] and proteomic [37,38,39,40,41] sequencing. The whole size of *J. curcas* was calculated as about 410 Mb and approximately 286 Mb consisting of 120,586 contigs and 29,831 singlets have been sequenced. These databases are valuable for further studies focusing on all aspects of molecular mechanisms. As an arisen economic woody species, *J. curcas* has been paid worldwide attentions. However, low temperature is still the major factor limited its distribution and affects the production of *J. curcas* seeds consequently. In consideration of the research and application, it is valuable to explore the metabolic network and regulated signal pathway of *J. curcas* under low temperature stress. Nevertheless, only few studies focused on molecular mechanism of chilling response in *J. curcas*. A previous study had employed transcriptome to analyze *J. curcas* under cold stress (12 °C), however, the result was sweeping without accurate mapping the regulated network basing on the experimental data, besides, the treatment temperature was also too moderate [42] to evaluate almost the limiting adverse effect under chilling stress. Another study only identified 8 photosynthesis related proteins significantly changed from *J. curcas* seedling under cold stress (4 °C) instead of high-throughput identification [43]. In this study, quantitative phosphoproteomics combining with traditional cellular observation, physiological measurement were employed to explore the chilling response and defense mechanism in *J. curcas* seedling at phosphorylation level. We aim to understand how sensitive is *J. curcas* to chilling stress and further unveil the specific phosphorylated proteins involved in potential pathways in *J. curcas* under chilling stress. The results will be benefit for providing clue to screen the chilling resistant *J. curcas* species.

## 2. Results

### 2.1. Physiological Changes of Leaves from J. curcas Seedling under Chilling Stress

**Figure 1:**
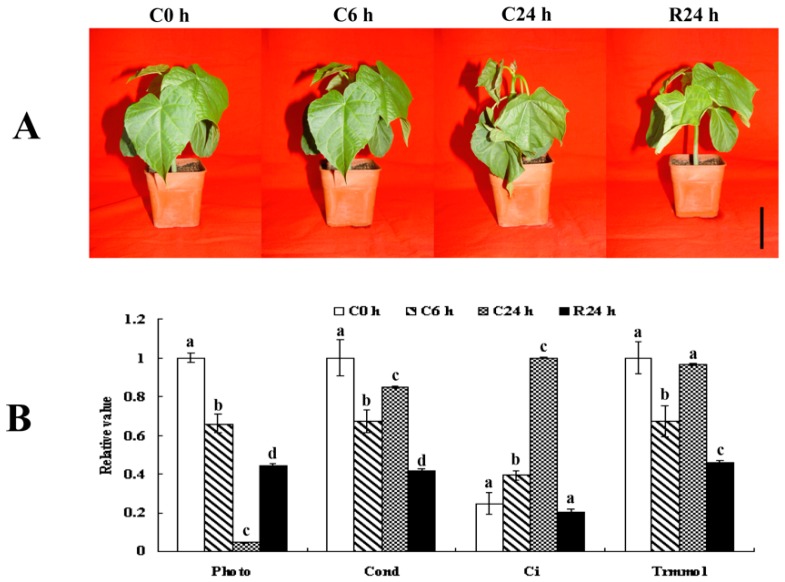
Morphological and physiological responses of *J. curcas* seedling to different treatment condition. Four-leaf stage seedling were treated at 4 °C for 0, 6 and 24 h (C0 h, C6 h and C24 h) and then allowed to recover for 24 h (R24 h) (**A**). The black bar equals 6 cm. The Pn, Cond, Ci and Trmmol were showed in (**B**). The values of relative % for each column are means ±S.D. of three biological replicates. The different lowercase letters labeled above columns indicate significant changes according to one-way ANOVA (*p* < 0.05).

The leaves of *J. curcas* seedling drooped and pseudostem tilted after 24 h of chilling treatment (4 °C) and mostly recovered after being returned to 28 °C for 24 h (Figure 1A). To evaluate the adverse effects of chilling stress quantitatively and determine the best time for sample collection after chilling stress for subsequent phosphoproteomic analysis, photosynthetic characteristics were measured. In response to the chilling treatment, the value of net photosynthetic rate (Pn) at each treatment stage (C0 h, C6 h, C24 h and R24 h) displayed significant difference with each other. the Pn decreased to 65.9% and 4.5% of pre-treatment (C0 h) levels after chilling for 6 h (C6 h) and 24 h (C24 h), respectively but returned to 44.3% after recovery for 24 h (R24 h). Comparing to Pn, the intercellular CO_2_ concentration (Ci) showed the opposite change tendency, the C6 h and C24 h of Ci increased to 160% and 408% respectively and almost returned to C0 h level after 24 h recovery. Conductance to H_2_O (Cond) and transpiration rate (Trmmol) shared the similar change curve, only the R24 h of Cond and Trmmol showed the significant changes compared with C0 h (Figure 1B). These findings were slightly different with the studies for *O. sativa* and *Musa paradisiaca* under chilling stress (4 °C), the species also originated in the tropics [44,45].

### 2.2. Ultrastructure Change of J. curcas Seedling under Chilling Stress

**Figure 2:**
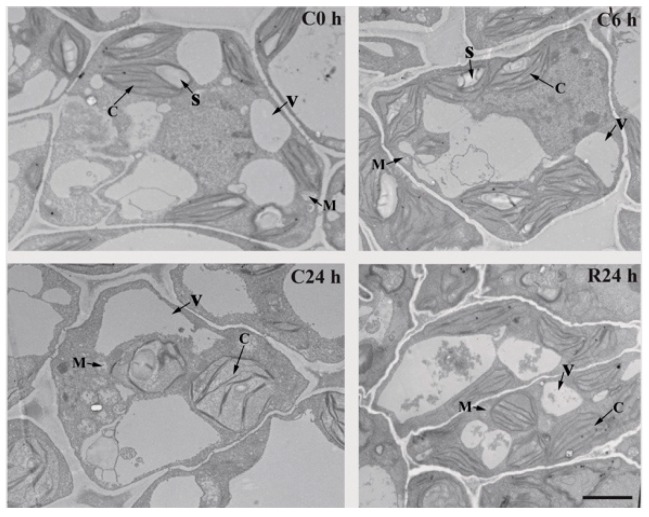
The ultrastructures of leaves at different treatment condition (C0 h, C6 h, C24 h, R24 h) from *J. curcas* seedling. The bar equals 2 μm. C, chloroplast; M, Mitochondria; S, starch granules; V, vacuole.

To assess the damage of *J. curcas* seedling under chilling treatment, the fourth-leaves at each treatment (C0 h, C6 h, C24 h and R24 h) were prepared for ultrastructural observation. In the leaf cell from C0 h, the morphological structures were intact chloroplasts with numerous embedded starch granules, which mainly distributed closely to the cell membrane. Besides, intact vacuole and mitochorina could also be observed clearly (Figure 2, C0 h). As a universal symptom, the obvious manifestations of chilling stress were chloroplast swelling, a distortion of vacuole, a reduction in the size of starch granules (Figure 2, C6 h), prolonged chilling treatment leaded to grana unstacked and starch granules continued to diminish with time till disappeared completely (Figure 2, C24 h). However, the mitochorina from the *J. curcas* seedling showed relative stationary and was not as sensitive as chloroplast and vacuole in the whole chilling treatment process (Figure 2). After recovery for 24 h, the chloroplasts of *J. curcas* seedling returned to normal morphological status (Figure 2, R24 h).

### 2.3. Phosphoprotein Identification and Phosphorylated Site Location

In total, 3101 phosphopeptides with 3101 phosphorylated sites corresponding to 1810 phosphoproteins were identified (Table S1) and the proportions of pS, pT and pY sites were calculated as 89.4%, 9.8% and 0.8%, respectively (Figure S1). The mass spectrometry proteomics data have been deposited in the ProteomeXchange Consortium (http://proteomecentral.proteomexchange.org) via the PRIDE partner repository [46] with the dataset identifier PXD011438. In order to evaluate analytical reproducibility, a range of quality control measures were taken for the three biological replicates of each condition before the comparison analysis of the phosphorylation levels between the chilling stress condition and the control. The result of Pearson correlation analysis showed that the four samples were individually clustered confidently with their replicates (Figure S2). Only the phosphopeptides identified from all biological repeats were used for further analysis.

### 2.4. Screening Phosphoproteins with Phosphorylation Level Significantly Changed

**Figure 3:**
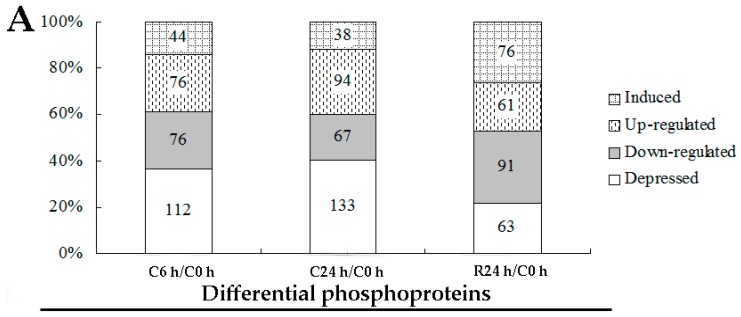

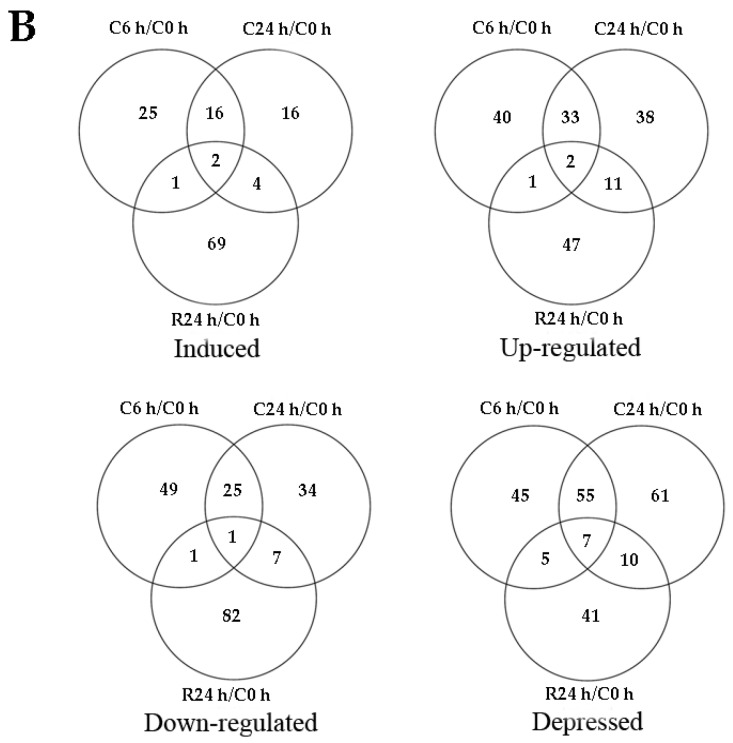
Statistical analysis of phosphoproteomics of *J. curcas* seedling under different treatment. The significantly changed phosphopeptides in each sample (C6 h, C24 h and R24 h) when compared to C0 h (**A**); Venn diagram of significantly changed phosphorylation sites and phosphoproteins distributed in each sample (C6 h, C24 h and R24 h) when compared to C0 h (**B**).

The intensity of each phosphopeptide was normalized as the Zhang et al. described [28]. According to the ANOVA analysis, 996 phosphorylated sites corresponding to 805 phosphoproteins were screened out (Table S2). Compared to untreated *J. curcas* seedling (C0 h), 308, 332 and 291 phosphorylation sites corresponding to 279, 313 and 270 phosphoproteins were differentially changed after chilling for 6, 24 h (C6 h, C24 h) and recovery for 24 h (R24 h), respectively (Tables S3–S5 and Figure 3A). There were 44, 76, 76 and 112 phosphorylation sites corresponding to induced, up regulated, down regulated and depressed regulation after 6 h chilling treatment, prolonged the chilling treatment time to 24 h, the number of phosphorylation site for each regulation has changed, which are 38, 94, 67 and 133, respectively (Figure 3A). To be mentioned, when recovery for 24 h, the number of induced phosphorylation sites increased obviously to 76 while the depressed ones decreased to 63 (Figure 3A). Further venny charts indicated that a number of differential phosphorylation sites were overlapped between C6 h and C24 h when compared to C0 h, however, only few differential phosphorylation sites were overlapped no matter the comparison between C6 h and R24 h or C24h and R24 h (Figure 3B).

### 2.5. GO Annotation of Significant Changed Phosphoproteins

**Figure 4:**
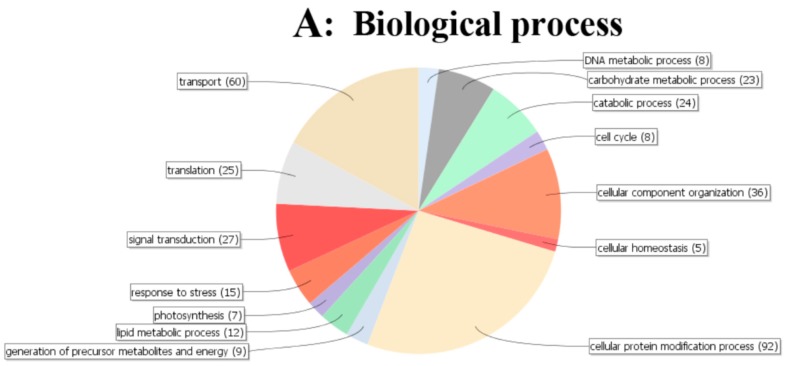

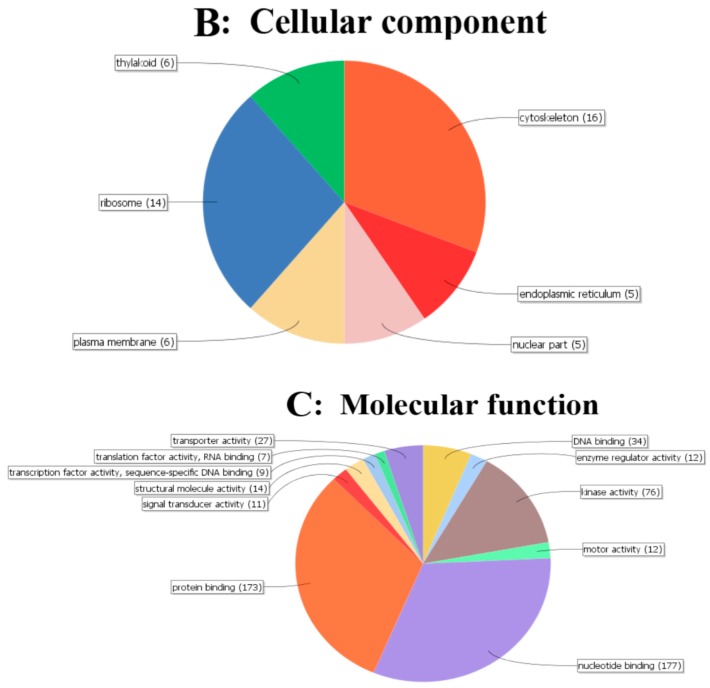
Functional classification of the significantly changed phosphoproteins from *J. curcas* seedling under different treatment by GO analysis. Over-represented GO terms were displayed graphically as pie charts for three GO vocabularies: (**A**) biological process; (**B**) cellular component; (**C**) molecular function. The number in brackets represents the phosphoprotein number within the group and the color of the pie chart represents the significance of enrichment.

All the identified significant changed phosphoproteins (805) were used for GO annotation. The distribution pie charts for biological process, cellular component and molecular function are shown in Figure 4. In the whole treatment process, the cellular protein modification process, transport, cellular component organization and signal transduction were significantly overrepresented from the biological process perspective. Cytoskeleton and ribosome were significantly overrepresented from the cellular component perspective. Nucleotide binding, protein binding and kinase activity were significantly overrepresented from the molecular function perspective (Figure 4).

### 2.6. Conservation Analysis of the Significant Changed Phosphoproteins

**Figure 5:**
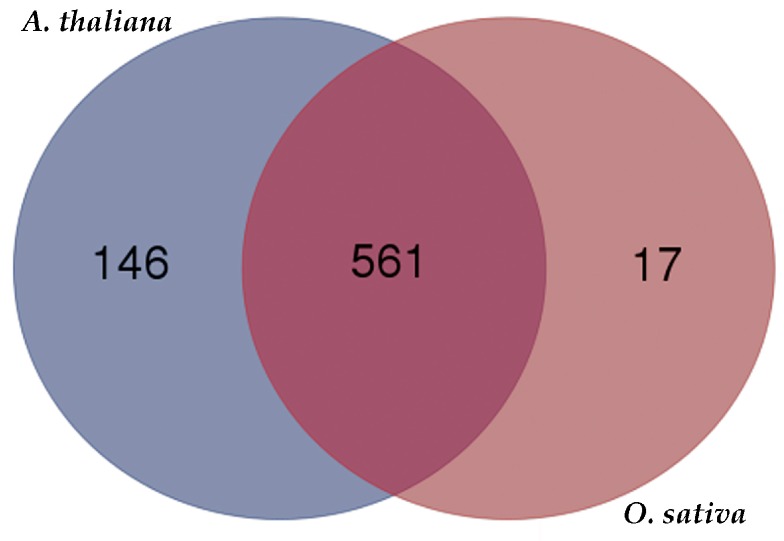
Conservation analysis of significantly changed phosphoproteins in *J. curcas* seedling under different treatment.

The sequences of the 805 significant changed phosphoproteins were used as queries to blast phosphoprotein databases that were constructed using data sets in the Plant Protein Phosphorylation DataBase (P3DB) [47] and PhoPhAt [48]. *O. sativa* and *A. thaliana* were compared against *J. curcas* to determine the degree of conservation of phosphorylation sites among different plant species. The thresholds were set as score ≥ 80, E-value < 1 × 10^−10^ and identity ≥ 30%. In all, 561 (69.7%) of the 805 phosphoproteins had phosphorylated orthologs in the two species, 163 (20.2%) had phosphorylated orthologs in only one species (Figure 5 and Table S6), 81 phosphoproteins had no phosphorylated orthologs in both of the two species (Table S6).

### 2.7. Analysis of Phosphorylation Motifs of Significant Changed Phosphopeptides

**Figure 6:**
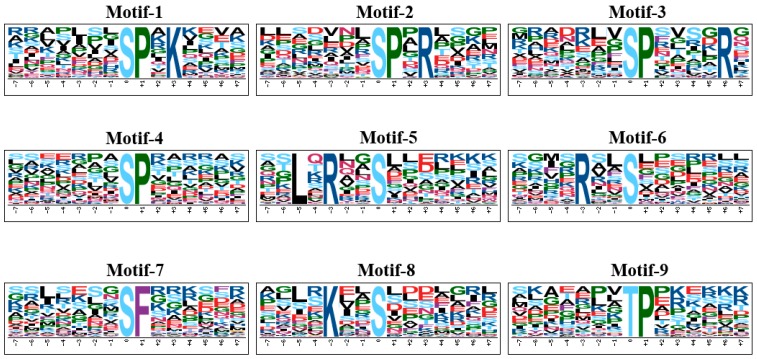
Phosphorylation motifs extracted from the phosphopeptides with the significantly changes by Motif-X from *J. curcas* seedling under different treatment.

The kinase related phosphorylation motifs of the significantly changed phosphopeptides were identified by employing WebLogo and motif-X. Briefly, the significant changed phosphopeptides were centered at the phosphorylated amino acid residues of each experimental group (Table S7) and then were submitted for Weblogo analysis and phosphorylation motif extraction. Nine phosphorylation motifs were enriched from the four experimental groups (Figure 6). Those phosphorylation motifs were then searched in relevant databases to find the specific protein kinases. [sPxK] and [sPxR] were the CDK motifs [49], [sPxxxxR] motif resembles the sPxR motif which is recognized by CDK [50,51]. [sP] and [tP] motifs were the proline-directed motifs, which were potential substrates of MAPK [49]. [LxRxxs] was basic motif representative of CDPK substrate and the motif [Rxxs] was a potential substrate for CDPK-II, which was also the 14-3-3 binding motif [52,53,54]. The two motifs [Rxxs] and [Kxxs] could be assigned to motif [-(K/R)-x-x-(pS/pT)-] and were identified as a phosphorylation motif of the SnRK2 or CDPK in plants by previous study [55]. [sF] contained the phenylalanine residue and was the minimal MAPK target motif [30].

### 2.8. Protein-Protein Interaction (PPI) Analysis of Significant Changed Phosphoproteins

**Figure 7:**
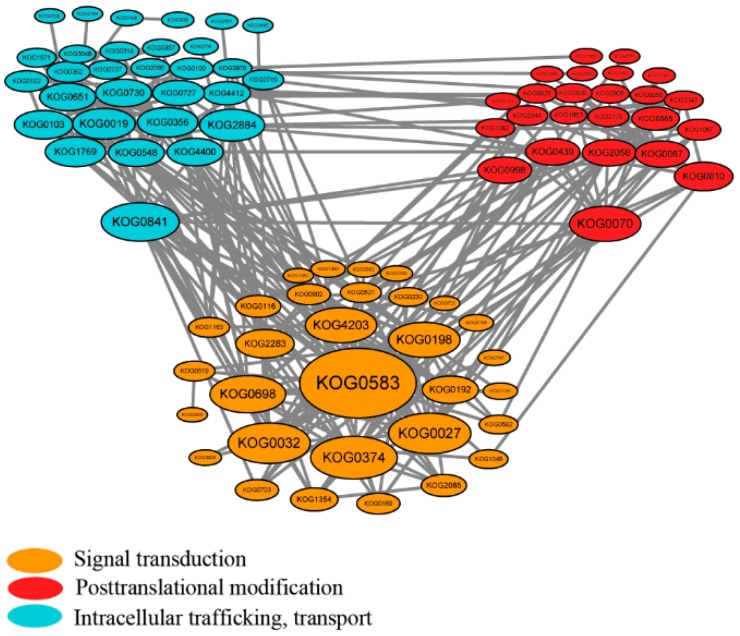
Protein-protein interaction (PPI) network of signal transduction, posttranslational modification and intracellular trafficking, transport related phosphoproteins by STRING. Nodes with orange, red and blue background color represent the KOGs of differential phosphoproteins related with signal transduction, posttranslational modification and intracellular trafficking, transport, respectively.

The PPI network of the significant changed phosphoproteins identified in the current study were analyzed by STRING (http://string-db.org, version 9.1). A total of 514 KOGs representing 610 phosphoproteins (Table S8) were used to construct the PPI network. In order to improve the reliability of the PPI analysis, the confidence score was set at the highest level (≥0.900). Finally, a complex PPI network that contained 319 nodes and 1924 edges was displayed through Cytoscape (Figure S3). With the aim to further extract the key potential interacting proteins from the whole PPI network, 85 KOGs representing 183 significantly changed phosphoproteins, which related to signal transduction, posttranslational modification and intracellular trafficking, transport (Tables S9 and S10), were chosen and centered to construct the subnetwork (Figure 7). The result showed that KOG0583 (protein id: 897 and 2462), KOG0841 (protein id: 1724 and 2765) and KOG0070 (Protein id: 5) were the centered phosphoproteins in each functional group (Table S9 and Figure 7).

### 2.9. An Overview of Response and Defense Mechanisms of J. curcas Seedling under Chilling Stress

**Figure 8:**
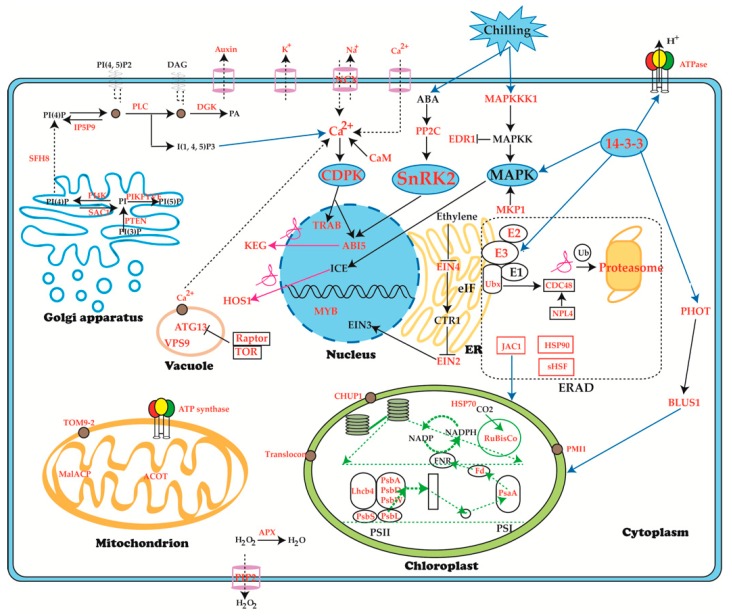
Schematic presentation of systematic chilling response and defense mechanisms in *J. curcas* seedling at phosphorylation level. The phosphorylation regulatory sites were shown in Table 1. Black solid lines with arrows represent catalytic reaction, while dotted lines with arrows represent material transport. Blue solid lines with arrows represent direct relationship, while magenta lines with arrows represent ubiquination. Black solid lines with stubs represent negative regulation. Green dotted lines with arrows in chloroplast represent transport between PSII and PSI. The proteins labeled with red colors represent significantly changed phosphoproteins among C0 h, C6 h, C24 h and R24 h. The area outlined with dotted square represents ER associated degradation (ERAD).

Based on the above results, 111 phosphoproteins (Table 1) with significant changes (with credible ANOVA analysis and fold change ≥ 2) were chosen to figure out a systematic chilling response and defense pathway in *J. curcas* seedling (Figure 8). The ion stress signal was transferred by chilling sensors on plasma membrane into cells and led to increase of Ca^2+^. Subsequently, ABA, ethylene, MAPK, Phosphatidylinositol, CDPK and 14-3-3 signal pathways were activated by phosphorylation modification in *J. curcas* seedling under chilling stress. These signals were then transferred into nucleus and induced the expression changes of response and defense related genes. Under chilling stress, photosynthesis was depressed, which resulted in the proteins associated with PSI and PSII to be significantly changed at the phosphorylation level. Channels and transporters on the membrane which were associated with ion, auxin, H_2_O_2_ also regulated through phosphorylation or dephosphorylation. According to change at phosphorylation level, the misfolded proteins were possibly handled either by refolding or degradation. Therefore, an ubiquitination mediated degradation pathway centered on E3s was displayed in the schematic representation. 

## 3. Discussion

We performed a comprehensive analysis of *J. curcas* seedling under chilling stress by employing the phosphoproteomic approach. Phosphorylation changes frequently occurred under chilling stress and the number of significantly changed phosphoproteins continually increased within the chilling duration (Figure 3 and Tables S2–S5). As a low temperature-sensitive species, *J. curcas* appeared to respond to and defend against chilling stress mainly through reversible protein phosphorylation, which was stimulated by Ca^2+^, phytohormones and phosphoinositide. The whole regulated network illustrated in this study mainly included signal transduction, ion transport and protein ubiquitination. At the early chilling treatment period, protein phosphorylation might involve in the regulation of ion transport for homeostasis. Prolonging the time of chilling treatment, the physiological injury appeared, which resulted in the proteins related chloroplast movement were phosphorylated against chilling (Figure 2 and Table 1). 

### 3.1. SnRKs Played a Central Role in the Chilling Responsive Signal Pathways 

When plants were exposed to chilling stress, the osmotic stress mediated by ABA accompanied and the corresponding signal pathways were triggered [4]. According to the PPI analysis (Figure 7), KOG0583 represented SnRKs (Table S9, protein id: 897 and 2462) showed the central position, which involved in the ABA mediated signal pathway and seemed to act as a vital defense approach against chilling stress in *J. curcas* seedling. JcSRK2a (id 2462) were observed to be significantly up-regulated phosphorylated in the chilling treatment process and return to normal condition at the recovery process, which might indicate that the JcSRK2a phosphorylation was activated by osmotic stress caused by chilling treatment. Multiple sequence alignments between JcSRK2a and 10 subfamily of SnRK2 from *A. thaliana* showed the JcSRK2a shared the highest similarity (86.44%) with AtSnRK2.4 which could be activated by osmotic stress [56]. Furthermore, overexpression of *TaSnRK2.4* in *A. thaliana* enhanced the plant tolerance to drought, salinity and low temperature [57]. The JcSRK2a (KDP38831.1) shared 79% homology with TaSnRK2.4 (ACU65228.1) and the phosphorylation site Ser348 of JcSRK2a was relative conserved comparing to the site of TaSnRK2.4 (Figure S4). These results provided a possible clue for us to improve low temperature resistance of *J. curcas*. In other hand, as the member of the SnRK1 subfamily [58], KIN10 is one of central transcriptional integrators of stress and energy signaling [59,60]. We observed KIN10 (id 897) was also observed to be phosphorylated changed as the SnRK2a showed. In consideration of these results, we hypothesized the phosphorylation of SnRK2a and KIN10 mainly involved in defense against chilling stress and promoting catabolism respectively in *J. curcas* seedling.

**Figure 9:**
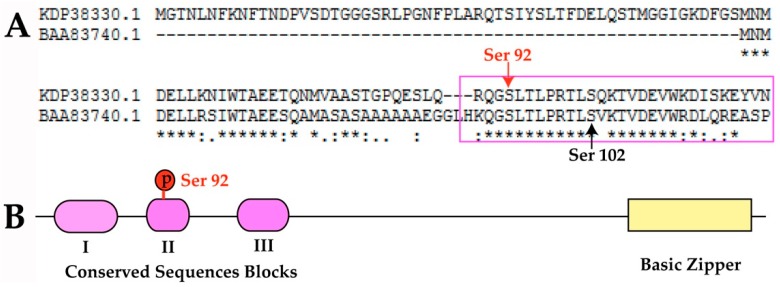
Sequence alignment and predicted structural composition of TRAB1 in *J. curcas*. (**A**) JcTRAB1 (KDP38330.1) sequence alignment with OsTRAB1 (BAA83740.1). The red line with arrow indicated the phosphorylation site of TRAB1 in *J. curcas* while black line with arrow indicated the phosphorylation site Ser 102 in *O. sativa*. The pink rectangle showed the conserved region II of TRAB1. (**B**) Predicted functional domain distribution of JcTRAB1.

ABI5 was highly inducible by either ABA treatment or stress [61]. In the presence of ABA, ABI5 accumulation was promoted by inducing KEG degradation, which might be accomplished by KEG self-phosphorylation [62]. In this study, the phosphorylation level of ABI5-2 (id 58) shared the similar change pattern as the SRK2a (id 2462) displayed (Figure S5), which might suggest the phosphorylation of ABI5-2 and SRK2a were both triggered by ABA. Additionally, ABI5 also underwent sumoylation at K391 residue to protect it from degradation [19], thereby possibly accelerating ABA-mediated growth inhibition in *J. curcas* seedling under chilling stress. Coincidently, as a negative regulator, JcKEG (id 2703) showed down regulation trend at phosphorylation level, which possibly indicated that ABA had induced KEG degradation. This assumption was supported by previous studies [18,62]. In response to ABA, TRAB1 phosphorylation at Ser 102 was essential and the TRAB1 phosphorylation level increased in a very short period and declined thereafter in *O. sativa* [16,63]. The JcTRAB1 (id 2400) phosphorylation level changed in a similar trend with OsTRAB1 (Table 1). Therefore, we compared the phosphorylation site of JcTRAB1 (KDP38330.1, id 2400) with OsTRAB1 (BAA83740.1). The result showed the phosphorylation site of JcTRAB1 was different with OsTRAB1, however, these two phosphorylation sites were very-close and both of them were in the conserved region II (Figure 9). Moreover, the phosphorylation motifs of JcTRAB1 and OsTRAB1 were [RxxS] and [LxRxxs] respectively, which were potential substrates of SnRK2 or CDPK in plants [55]. It was assumed the JcTRAB1 phosphorylation at Ser in this conserved region was possibly related to the ABA response.

The neighbors of SnRKs were chilling stress related protein kinases, which participated in multiple biological processes. Cytosolic Ca^2+^ transiently increased as the result from chilling stress [64]. CDPK played a key role in abiotic stress and mediated in Ca^2+^ signal transduction [65]. The phosphorylation of OsCDPK13 had been indicated as an important signal event in response to cold stress [66]. In this study, JcCDPK8 (id 290) was inducible phosphorylated at Ser 43 only under chilling stress (Table 1), which might indicate that JcCDPK8 functioned in the phosphorylation status in response to chilling stress. In ethylene mediated signal pathway, ethylene responses were negatively regulated by receptors family [67]. Two proteins EIN4 (id 521) and EIN2 (id 1204) were phosphorylated changed. EIN4 and EIN2 were responsible for the negative and positive regulations respectively in ethylene mediated signal [68]. Although it was unclear whether the protein expression level of EIN4 and EIN2 had changed, their changes at phosphorylation level might suggest they were likely related to the ethylene signaling. Additionally, phosphorylation changes were also implicated in MAPK mediated signaling. Phosphorylated proteins involved in MAPK signaling including EDR (id 2304), MKP1 (id 2287) and HOS1 (id 2597) were all significantly changed at phosphorylation level. MAPKs were implicated in response to low temperature condition [69,70,71,72], as well as interacted with MKP1 [73]. In this study, we did not detect the significantly change of MAPK at phosphorylation level. However, MKP1 (id 2287) showed an elevated phosphorylation level under chilling treatment (id 2287), suggesting a possible link with MAPK function in *J. curcas*. MAPK subsequently interacted with ICE1, which was degraded by an E3 ligase HOS1 [21]. The phosphorylation level of HOS1 (id 2597) was increasing under chilling stress, possibly resulting in ICE1 degradation. It might be a possible reason to explain why no significantly changed ICE1 at the phosphorylation level was detected in this study.

### 3.2. Phosphorylation Participated in the Regulation of Phosphoinositide Metabolism

As the primary lipid-derived signals, phosphoinositides were involved in various plant responses to surrounding environment [74]. A total of 13 participants in phosphoinositide metabolism were differentially phosphorylated in this study (Table 1). They participated in reactions leading to the generation of different phosphoinositide species. Among them, PLC2 was the decisive enzyme in phosphoinositide metabolism [75], it catalyzed the hydrolysis of PI(4,5)P2 into DAG and IP3, which subsequently acted as two important second messenger molecules and resulted in release of Ca^2+^ into the cytoplasm [76]. Recent studies showed the expressions of *JcPLC2* and *BpPLC2* were constitutive even under low temperature stress [27,77]. In *J. curcas* seedling, PLC2s (id 3110, 3180) were both differential phosphorylated at recovery stage instead of the chilling stage. Their phosphorylation sites were Ser312 and Ser278 respectively. The phosphorylation site Ser312 from PLC2 (KDP44118.1, id 3110) was located in the catalytic Y domain (Figure S6), which have been reported in AtPLC2 (NP_187464.1) [78,79]. It possibly suggested the regulation of PLC2 activity by phosphorylation in *J. curcas* seedling at the recovery stage. Besides, the present study showed that PI4K (id 3364) was significantly and continuously up-regulated at the phosphorylation level under chilling stress and returned to normal level at the recovery stage (Table 1). PI4K was responsible for the generation of PI4P from PI and it showed important role in trafficking from the trans-Golgi network to the prevacuolar compartment [76]. Meanwhile, the phosphorylation level of DAGK 5-like (id 3551) showed the completely opposite trend with PI4K (Figure S5). In *A. thaliana*, PI4K was indicatively activated during cold stress [80]. In other hand, PI4P could be dephosphorylated by SAC1-domain phosphoinositide phosphatases [81]. As the revisable phosphatase, SAC1 (id 2911) was down regulated at phosphorylation level (Table 1). By means of the PI4K, DAGK and SAC1 changes at phosphorylation level under chilling stress, we assumed that there was a potential straightforward metabolic pathway for generation of IP3 and the phosphorylation event was seemed to be decisive for phosphoinositide metabolism in *J. curcas* seedling under chilling stress.

### 3.3. Detoxification and Stress Defense

Chilling caused H_2_O_2_ accumulation and H_2_O_2_ could be diffused by aquaporins in phosphorylation status at the plasma membrane in the response to chilling stress in maize [82]. Aquaporins were proteinaceous pores that facilitated passive water transport through membranes of living cells [83]. Three aquaporins were examined as the significantly phosphorylated proteins in this study. The phosphorylation level of PIP2-8 (id 1 and id 2) showed the identical change trends (Figure S5), they both increased at the chilling treatment stage and return to normal level after recovery for 24 h. However, another one (PIP2-5, id 3151) maintained constant phosphorylation level even under chilling stress and increased at the recovery stage (Table 1). This might suggest phosphorylation was able to influence the activity of aquaporins and potentially related to H_2_O_2_ diffusion in *J. curcas* seedling under chilling stress. On the other hand, as the typical antioxidant enzyme, APX1 catalyzed the H_2_O_2_-dependent oxidation of ascorbate in plants [84]. APX1 (id 15) was significantly up-regulated phosphorylated in response to chilling, which possibly indicated the APX1 phosphorylated change was related to H_2_O_2_ detoxification in *J. curcas* seedling. 

Serine threonine-protein kinase BLUS1 mediated a primary step for phototropin signaling in guard cells and it was directly phosphorylated by PHOT1. BLUS1 functioned as a PHOT substrate and primary regulator of stomatal control to enhance photosynthetic CO_2_ assimilation under natural light conditions [85]. BLUS1 (id 655) showed down regulated trends at phosphorylation level, which was corresponding to the phosphorylated change pattern of PHOT1 (id 3118) (Figure S5). Meanwhile, CDPK 33 was also involved in the ABA-mediated regulation of stomatal closure and drought stress responses [86]. The phosphorylation level of JcCDPK33 (id 2491) increased greatly after chilling for 6 h, CDPK33 contained the motif [sP] and potentially combined with MAPK [49], as well as possibly interacted with 14-3-3 [87]. 14-3-3 protein 6 (id 2765) also contained [sP] motif, which was dramatically up-regulated (2.7-fold) at phosphorylation level after chilling for 6 h (Table 1). It was hypothesized that phosphorylation of PHOT, CDPK and 14-3-3 signaling related proteins was possibly involved in stomatal closure in *J. curcas* seedling under chilling stress.

Under chilling stress, many proteins were misfolded and these proteins could not be assembled and function as normal. There were two approaches to handle these misfolded proteins. One was refolding in the assistance of molecular chaperones. In this study, CPN60B2 (id 1375) and CHSP70 (id 3984) were both phosphorylated changed and might result in the activation of RuBisCo assembly [88,89,90]. Nevertheless, the phosphorylation level of sHSP (id 2898), Hsp90 (id 1658) and PPI cyp65 (id 1583) showed little changes under chilling treatment. These results possibly indicate the accumulation of misfolded proteins in ER [91]. Another was degradation of the ubiquitinated misfolded proteins by the 26S proteasome. 26S protease regulatory subunit 6b homolog (id 1742) showed obviously phosphorylated change after chilling for 24 h and remained the phosphorylation level after 24 h recovery (Table 1). We speculated that the misfolded proteins in *J. curcas* seedling were mainly handled through degradation by ubiquitin-proteasome system at continuous chilling (24 h) and recovery period. 

### 3.4. Photoinhibition and Chloroplast Movement

In response to the chilling stress, changes could be observed at the physiological and cellular level in *J. curcas* seedling within 24 h chilling treatment (Figure 1; Figure 2), which was consistent with the previous description [5]. Chloroplast was the first and most severely affected organelle when plants were exposed to freezing stresses. The photosyntic rate of *J. curcas* seedling was dramatically decreased (Figure 1B) and the chloroplasts were swelling after 24 h chilling treatment (Figure 2), which indicated serious damage of the photosystem in *J. curcas* seedling. At chilling temperature, it was well known that photoinhibition of photosynthesis was enhanced [92] and the primary target of photoinhibition was PSII [93] and phosphorylation of PSII centers increased the stability of PSII complexes and concomitantly improved their survival under stress conditions [94]. In this study, as an important component of PSII, D1 (id 3541) was rapidly phosphorylated under chilling treatment for 6 h, which might result from an imbalance between energy supply and utilization in chloroplasts of chilling-sensitive *J. curcas* seedling. The rate of PSII repair was reduced by inhibiting D1 synthesis at the translation-elongation stage if the repair was not efficiently scavenged [95]. Coincidently, with the time of chilling treatment prolonged, the phosphorylation level of D1 dramatically decreased. It might suggest the phosphorylation of D1 was closely related to stability of PSII complexes. During recovery from chilling-induced photoinhibition in leaves of *J. curcas* seedling, we observed that the CP29 (id 3634) dephosphorylation matched very well with those of PSII recovery. These results might suggest that PSII reactivation from low temperature photoinhibition was closely related to CP29 dephosphorylation. Similar result had been illuminated at *O. sativa* during recovery from chilling induced photoinhibition [96].

Chloroplast movement was an efficient strategy to alleviate PSII damage and maintained maximal photosynthetic output [97]. In plants, temperature was an important factor in modulating chloroplast relocations and PHOTs were considered to mediate not only stomatal opening but also chloroplast movement [98]. The protein level of PHOT1 decreased while the PHOT2 slightly increased at low temperature in *A. thaliana* [99]. In this study, the phosphorylation level of PHOT1 (id 3118) was down-regulated while the PHOT2 (id 3490) was up-regulated after chilling treatment for 6 h and 24 h, which possibly indicated that phosphorylation related to the activity of PHOT1 and PHOT2. However, both of the PHOT1 and PHOT2 maintained the phosphorylation level previously even after the stimulus was removed (Table 1). Besides the PHOTs, the chloroplast movement was also associated with several proteins [100]. CHUP1 might function at the periphery of the chloroplast outer membrane and most likely represented an essential component for chloroplast movement. Similarly, the mutant *pmi1* exhibited severely attenuated chloroplast movements under low and high light intensities, indicating that PMI1 was necessary for both chloroplast accumulation and avoidance movements [101]. Nevertheless, we observed little change of CHUP1 (id 1231 and id 1233), PMI1 (id 2153 and id 2154) at the phosphorylation level after chilling treatment in *J. curcas* seedling, subsequently, all of them inducible regulated at the phosphorylation level in the recovery stage. To date, there were relatively few physiological and molecular evidences for explaining chloroplast movement through phosphorylation. Therefore, we speculated that the activities of CHUP1 and PMI1 might be regulated by other post-translational modification, such as glycosylation or sumoylation. 

## 4. Materials and Methods 

### 4.1. Plant Material and Chilling Treatment

The uniform mature *J. curcas* seeds with same provenance were collected from Xishuangbanna Tropical Botanical Garden, Chinese Academy of Sciences, Yunnan Province. The mature *J. curcas* seeds were sowed and grown in a mixed soil of peat and vermiculite (1:1) in a growth chamber under 28/25 °C (day/night) temperature regime, a photon flux density of 150 μmol m^−2^·s^−1^ throughout a 14-h photoperiod and a relative humidity of 80%. Four-leaf stage seedlings were used in this experiment. Low temperature treatments were started at 11:00 AM on the first day by setting the temperature to 4 °C, which was reached about 50 min later. The temperature was returned to 28 °C after 24 h of chilling treatment. The fourth true leaves were detached from the top of *J. curcas* seedling at each time point (4 °C for 0, 6, 24 h and then returned to 28 °C for 24 h), which indicated as C0 h, C6 h, C24 h and R24 h respectively for each biological replicate. one part of the collected leaves were used for tissue preparation of transmission electron microscopy and other part of the collected leaves from the five plants were frozen in liquid nitrogen and stored at −80 °C until use. However, in consideration of the functional maturity, the first true leaves were chosen for measurements of photosynthesis, stomatal conductance and transpiration. The measurement was carried out by using a portable gas analysis system, LI-COR 6400 with a light-emitting diode light source (LICOR Inc., Lincoln, NE, USA). The measurement conditions were set as follows: block temperature 20 °C, photon flux density 1200 μmol m^−2^·s^−1^, humidity 80% and the CO_2_ concentration was stabilized by 5 L surge flask. 

### 4.2. Tissue Preparation for Transmission Electron Microscopy and Protein Preparation 

The fourth true leaves from C0 h, C6 h, C24 h and R24 h were fixed in 2.5% glutaraldehyde in 100 mM phosphate buffer (pH 7.0) for 4 h at room temperature. Then the samples were handled as the previous operation [38]. Leaf total protein was extracted according to the method of Shen et al. [102] with minor modifications. Approximately 500 mg leaves of each sample was homogenized in 2 mL of the homogenization buffer containing 20 mM Tris-HCl (pH 7.5), 250 mM sucrose, 10 mM ethylene glycol tetra acetic acid, 1mM phenylmethylsulfonyl fluoride, 1% dithiothreitol (DTT) and 1% Triton X-100 on the ice. The homogenate was collected into an Eppendorf tube and centrifuged at 10,000× *g* for 10 min at 4 °C. The supernatant was transferred to a fresh tube and precipitated by adding 10% cold trichloroacetic acid on ice for over 30 min. The mixture was centrifuged at 15,000 g for 10 min at 4 °C and the supernatant was discarded. After washed three times with acetone containing 1% DTT, the pellet was collected by centrifugation, air-dried and then suspended in SDT buffer containing 4% SDS, 1 mM DTT, 100 mM Tris-HCl (pH 7.5), sonicated for 2 min (with cooling on ice) and boiled for 15 min. The protein mixtures were harvested via centrifugation at 15,000× *g* for 20 min at 4 °C to remove insoluble material. Protein concentrations of experimental samples were quantified according to Bradford method [103]. Albumin (A5503, Sigma, St. Louis, MO, USA) was used as a standard for protein quantification. Three independent biological replicates were performed independently for each experimental sample.

### 4.3. Phosphopeptide Enrichment

The same amount of extracted protein mixture in each sample was directly reduced with DTT, alkylated with iodoacetamide and subsequently digested with endoproteinase Lys-C and trypsin as previously described [104]. The enrichment procedure for the phosphopeptides was performed as followed. Tryptic peptides (5 mg) were dissolved in 400 μL of loading buffer containing 65% acetonitrile (ACN)/2% trifluoroaceticacid (TFA) saturated with glutamic acid and incubated with an appropriate amount (tryptic peptide: TiO_2_ = 1:1, *w*/*w*) of TiO_2_ beads (GL Sciences, Tokyo, Japan) for 40 min. The mixture was centrifuged at 10, 000× *g* for 3 min at 4 °C and the supernatant was discarded. After washing with 800 μL wash buffer (65% ACN/0.1% TFA) for 40 min, then centrifugation as above, then the phosphopeptides were eluted twice with 800 *μ*L elution buffer (500 mM NH_4_OH/60% ACN), centrifugation and the eluate was dried and reconstituted in 0.1% formic acid/H_2_O for MS analysis.

### 4.4. LC-MS/MS Analysis

The enriched phosphopeptides were separated on a self-packed C18 reverse-phase column (70 μm inner diameter, 150-mm length, Column Technology, Fremont, CA, USA) that directly connected to the nanospray ion source to an Q Exactive mass spectrometer (Thermo Fisher Scientific, San Jose, CA, USA) running in the positive ion mode. The pump flow was split to achieve a flow rate of 1 μL/min for sample loading and 300 nL/min for MS analysis. The mobile phases consisted of 0.1% formic acid (A) and 0.1% formic acid and 90% ACN (B). A four-step linear gradient of 2% to 5% B in 5 min, 5% to 22% B in 55 min, 22% to 90% B in 5 min and keeping 90% B for 10 min. The spray voltage was set at 2.0 kV and the temperature of the heated capillary was 270 °C. For data acquisition, each MS scan was acquired at a resolution of 60,000 (at 400 *m*/*z*) with the lock mass option enabled. A lock mass function was used to obtain high mass accuracy. The 12 most intense precursor ions were selected for collision-induced fragmentation in the linear ion trap at normalized collision energy of 37%. The threshold for precursor ion selection was 500 and the mass window for precursor ion selection was 2.0 Da. The dynamic exclusion duration was 120 s, the repeat count was 1 and the repeat duration was 30 s. Three biological replicates were performed independently from sample collection to the phosphopeptide identification using LC-MS/MS.

### 4.5. Protein Identification

The raw files were processed with MaxQuant (version 1.3.0.5) and searched against the NCBI Jatropha protein database (update to 20180509, 41287 entries) concatenated with a decoy consisting of reversed sequences. The following parameters were used for database searches: trypsin/P was chosen as enzyme specificity, Carbamidomethyl (C) was selected as a fixed modification, Oxidation (M), Acetyl (Protein N-term) and Phospho (STY) were selected as variable modifications. Up to two missing cleavage points were allowed. The precursor ion mass tolerances were 7 ppm and the fragment ion mass tolerances for the MS/MS spectra were 20 ppm. The false discovery rate (FDR) was set to ≤0.01 for peptide and protein. The minimum peptide length was set to 6. The localization of the phosphorylation site was based on the PTM scores that assigned probabilities for each of the possible sites according to their site-determining ions. The software MaxQuant was used to calculate the PTM scores and PTM localization probabilities. Only when the phosphorylation site of localization probabilities (*p* ≥ 0.75) and the PTM score (≥5) was considered as the reliable. An FDR of 0.01 was used for phosphorylation site identification.

### 4.6. Label Free Quantification and Screening of Phosphopeptides with Significant Changes at the Phosphorylation Level

We integrated the ion intensities over its chromatographic elute profile and employed MaxQuant software to calculate the quantification of phosphopeptides on the basis of a label free approach. For each phosphopeptide, its intensity was normalized to the mean of the intensities of all phosphopeptides within each biological replicate. The screening terms of phosphopeptides with significant changed at phosphorylation level were listed as below: (1) phosphopeptides detected in all three biological replicates; (2) phosphopeptides with credible ANOVA analysis or Student’s-test analysis (FDR<0.05); (3) phosphorylation localization probability ≥ 0.75 and (4) phosphorylation site score difference ≥ 5.

### 4.7. Bioinformatics

Protein function was annotated through Blast2GO software (http://www.blast2go.com/b2ghome), significantly enriched phosphorylation motifs were extracted from phosphopeptides with confidently identified phosphorylation residues using the Motif-X algorithm (http://motif-x.med.harvard.edu/). The phosphopeptides were centered at the phosphorylated amino acid residues and aligned and seven positions upstream and downstream of the phosphorylation sites were included. Because the upload restriction of Motif-X is 10 Mb, a FASTA format data set (nearly 10 Mb) containing the protein sequences from the *J. curcas* protein database was used as the background database to normalize the scores against the random distributions of amino acids. The occurrences threshold was set to 5% of the input data, set at a minimum of 20 peptides and the probability threshold was set to *p* < 10^−6^. The phosphoproteins blasted by the National Center for Biotechnology Information (NCBI) were used to obtain the KOG numbers of those proteins by eggNOG (http://eggnogdb.embl.de/). A data set containing all the KOG numbers was then used for PPI by using the Search Tool for the Retrieval of Interaction Genes/Proteins (STRING) database (http://string-db.org/) and the PPI network was displayed by Cytoscape software (Version 3.6.3).

## 5. Conclusions

In summary, the woody oil plant species, *J. curcas* seedling was sensitive to chilling stress. It was studied by employing a comparative phosphoproteomic analysis, physiological measurement, ultrastructure observation under chilling stress and recovery. For the 805 significantly changed phosphopeptides, 9 phosphorylation motifs were extracted, which were mainly regulated by CDK, SnRK2, MAPK and CDPK. A complex PPI subnetwork were constructed and indicated the crucial roles of SnRK, 14-3-3 and ADP-ribosylation factor in the responsive network. Our results showed the phosphorylation was an essential event and responsible for chilling response and defense in *J. curcas* seedling. Consequently, Ca^2+^, ABA, ethylene, phosphoinositide and 14-3-3 mediated signal pathways cross linked for chilling response. In response to chilling stress, the phosphorylation of SnRK2a, ABI5 and TRAB1 were possibly related to ABA mediated signal pathway. Additionally, the phosphorylation level of transport, photoinhibition and chloroplast movement related proteins were also significantly regulated. We also highlighted that the phosphorylation of JcHOS1, JcKEG, JcAPX, JcPIP2 and JcPI4K might activate the potential functions to defense against chilling stress. Finally, we depicted a schematic presentation including signal transduction, metabolism and ion transport on the basis of changes at phosphorylation level, which is valuable for us to understand the chilling response and defense network in *J. curcas* seedling. 

## Figures and Tables

**Table 1:** 110 significantly changed phosphoproteins from *J. curcas* seedling in response to chilling.

ID	Proteins	Annotation	Modified Sequence	Change Pattern *
**Ions and Transport**				
1	ABM54183.1	Aquaporin pip2-8	_ALGS(ph)FRS(ph)NPTN_	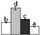
2	ABM54183.1	Aquaporin pip2-8	_ALGSFRS(ph)NPTN_	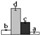
3151	KDP44363.1	Aquaporin PIP2-5	_VKDVAEQGS(ph)FSAK_	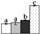
555	KDP24122.1	Two pore calcium channel protein 1	_(ac)M(ox)EKPLLGETSSNAS(ph)FR_	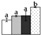
172	KDP21053.1	Sodium/calcium exchanger NCL2-like	_LVNDEGQVDVS(ph)CIKR_	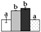
1832	KDP33816.1	K(+) efflux antiporter 4	_GDS(ph)FRADSAK_	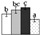
535	KDP23925.1	Auxin efflux carrier component 1c	_EDFS(ph)FGNR_	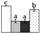
536	KDP23925.1	Auxin efflux carrier component 1c	_LAVS(ph)PGKVEGQR_	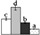
2342	KDP38049.1	Auxin efflux carrier component 3-like	_AIANAGDFPGEDFS(ph)FAGK_	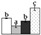
2343	KDP38049.1	Auxin efflux carrier component 3-like	_LGS(ph)SSTAELHPK_	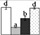
2352	KDP38049.1	Auxin efflux carrier component 3-like	_S(ph)LGPGS(ph)FSALTPR_	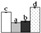
1468	KDP31195.1	ATPase plasma membrane-type	_(ac)GDKS(ph)EVLEAVLK_	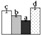
2710	KDP40607.1	ATPase plasma membrane-type	_GHVES(ph)VIR_	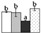
2711	KDP40607.1	ATPase plasma membrane-type	_S(ph)LQGLM(ox)AADLEFNGK_	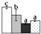
3274	KDP45466.1	Plasma membrane ATPase 1-like	_GS(ph)FNELNQMAEEAK_	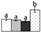
3755	KDP31195.1	ATPase plasma membrane-type	_T(ph)LHGLQPPDTK_	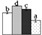
3794	KDP33445.1	Plasma membrane ATPase 4	_GLDIDTIQQHYT(ph)V_	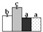
3836	KDP35383.1	Plasma membrane ATPase 4	_T(ph)LHGLQPPETASIFNEK_	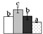
3941	KDP40607.1	ATPase plasma membrane-type	_NLDLNVIQGAHT(ph)V_	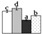
**Phosphorylation Events Involved in Photoinhibition**				
9	ACN72692.1	PsaA	_IIRS(ph)PEPEVK_	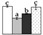
508	KDP23672.1	Photosystem II reaction center W chloroplastic-like	_LATILPAAS(ph)FK_	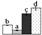
1690	KDP32802.1	Photosystem II 22 kDa chloroplastic	_FVDDPPTGIEGAVIPPGKS(ph)FR_	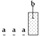
3541	ACN72673.1	photosystem II protein D1	_(ac)T(ph)AILERR_	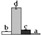
3542	ACN72687.1	photosystem II protein D2	_(ac)T(ph)IALGKFTK_	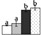
3544	ACN72707.1	photosystem II protein L	_(ac)T(ph)QSNPNEQNVELNR_	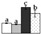
3634	KDP24461.1	Chlorophyll a-b binding protein CP29.1 chloroplastic	_T(ph)ELADVK_	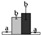
4027	KDP44781.1	Chlorophyll a-b binding protein CP29.3 chloroplastic	_FGFPGFGT(ph)KK_	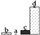
3900	KDP38815.1	Ferredoxin-A-like	_LLT(ph)PEGEK_	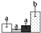
3091	KDP44002.1	Ru large subunit-binding protein subunit alpha	_NVVLDEFGS(ph)PK_	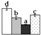
98	KDP20412.1	Translocase of chloroplast chloroplastic-like	_LVNGS(ph)SEDIR_	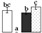
1008	KDP28139.1	Translocase of chloroplast chloroplastic	_M(ox)NEETEVLS(ph)GGNEK_	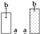
**Chloroplast movement**				
1231	KDP29354.1	Protein CHUP1, chloroplastic	_NAGETVAITS(ph)FGK_	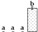
1233	KDP29354.1	Protein CHUP1, chloroplastic	_SFS(ph)GGSPR_	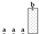
2153	KDP36752.1	Plastid movement impaired1	_LTELDS(ph)IAQQIK_	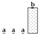
2154	KDP36752.1	Plastid movement impaired1	_MEDETES(ph)QRLDADEETVTR_	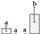
2419	KDP38475.1	J domain-containing protein required for chloroplast accumulation response 1	_VLS(ph)PGRPLPPR_	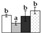
2248	KDP37156.1	Root phototropism protein 3	_S(ph)PNLGFEQPGGSISK_	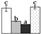
2767	KDP40950.1	Root phototropism protein 3	_LLEHFLVQEQTENSS(ph)PSR_	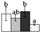
3298	KDP45678.1	Root phototropism protein 2	_NQLQTDVS(ph)LIR_	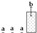
3118	KDP44148.1	Phototropin-1	_RNS(ph)ENVPSNR_	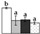
3490	KDP47050.1	Phototropin-2	_FAVDSTRTS(ph)EESEAGAFPR_	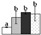
**ROS related**				
15	ACV50426.1	Cytosolic ascorbate peroxidase-1	_HS(ph)AELAHAANTGLDIALR_	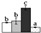
16	ACV50426.1	Cytosolic ascorbate peroxidase-1	_LPGANEGS(ph)DHLR_	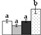
773	KDP26232.1	Pyrroline-5-carboxylate reductase	_DDVAS(ph)PGGTTIAGIHELEK_	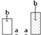
2948	KDP42714.1	Monodehydroascorbate reductase	_VIGAFLEGGS(ph)PDENQAIAK_	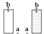
**CDPK related signal**				
133	KDP20751.1	Calcium-dependent protein kinase 21-like	_LGS(ph)KLSETEVK_	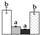
286	KDP21986.1	CDPK-related kinase 4-like	_WPLPPPS(ph)PAK_	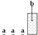
290	KDP22101.1	Calcium-dependent protein kinase 8-like	_ENPFFGNDYVVNNGS(ph)GR_	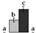
580	KDP24440.1	Calcium-dependent protein kinase 13	_FNSLS(ph)MK_	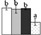
1253	KDP29499.1	Calcium-dependent protein kinase 1	_NS(ph)FSIGFR_	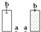
2491	KDP39132.1	Calcium-dependent protein kinase 33-like	_S(ph)PVQPTYQLPSQQPPIHVPR_	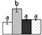
3476	KDP47014.1	Calcium-dependent protein kinase 26	_LYQGINQPEEQSAASHS(ph)K_	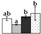
3477	KDP47014.1	Calcium-dependent protein kinase 26	_NS(ph)LNMSMR_	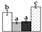
2413	KDP38457.1	Calcium-binding protein CML41	_LITSS(ph)LPR_	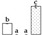
3546	KDP45998.1	Calmodulin-7	_M(ox)KDT(ph)DSEEELK_	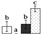
**Ethylene and ABA related signal**				
521	KDP23695.1	Protein EIN4	_VFSENGS(ph)EGKNDR_	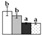
1204	KDP29130.1	Ethylene-insensitive protein 2	_M(ox)VPGSISSVTYDGPPS(ph)FR_	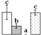
897	KDP27185.1	SNF1-related protein kinase catalytic subunit alpha kin10	_M(ox)HANEPTS(ph)PAVGHR_	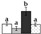
1412	KDP30923.1	SNF1-related protein kinase regulatory subunit gamma-1	_S(ph)PEANLGM(ox)K_	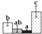
2036	KDP35506.1	SNF1-related protein kinase regulatory subunit beta-2	_GEEEGLNS(ph)PSGSGGGGIDGGGGGGGR_	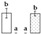
2462	KDP38831.1	Serine threonine-protein kinase SRK2a	_VKEAQAS(ph)GEFR_	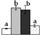
58	AIA57942.1	Abscisic acid-insensitive 5-like protein 2	_QAS(ph)LSLTSALSK_	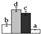
2400	KDP38330.1	bZIP transcription factor TRAB1	_QGS(ph)LTLPR_	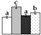
2703	KDP40550.1	E3 ubiquitin-protein ligase KEG	_VGFPGAS(ph)R_	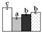
2644	KDP40139.1	Protein phosphatase 2c 70	_S(ph)GPEKDDLLESVPK_	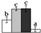
3925	KDP40139.1	Protein phosphatase 2C 70	_VGQT(ph)LKR_	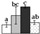
**MAPK related signal**				
1678	KDP32742.1	Mitogen-activated protein kinase kinase kinase 1	_FHDM(ox)DS(ph)PR_	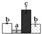
2287	KDP37397.1	Protein-tyrosine-phosphatase MKP1	_S(ph)LDEWPK_	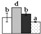
2304	KDP37750.1	serine/threonine-protein kinase EDR1	_SIS(ph)MTPEIGDDIVR_	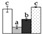
2597	KDP39907.1	E3 ubiquitin-protein ligase HOS1	_IS(ph)PSSLADR_	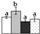
**Phosphoinositide metabolism**				
1558	KDP31989.1	Phosphatidylinositol-3,4,5-trisphosphate 3-phosphatase and dual-specificity protein phosphatase PTEN, putative	_LTS(ph)GFGLHFASAAPGPNESSK_	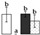
1873	KDP33967.1	1-phosphatidylinositol-3-phosphate 5-kinase Fab1a	_S(ph)FGSGEYR_	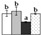
2004	KDP35146.1	Phosphoinositide phosphatase SAC9	_RAS(ph)FGGSVENDPCLHAR_	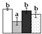
2372	KDP38175.1	type IV inositol polyphosphate 5-phosphatase 9	_GPS(ph)LDLPR_	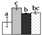
2718	KDP40633.1	1-phosphatidylinositol-3-phosphate 5-kinase Fab1b	_TSSS(ph)FGSGEFR_	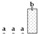
2735	KDP40719.1	Phospholipid:diacylglycerol acyltransferase 1-like	_S(ph)REPTNSSTDLK_	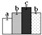
2911	KDP42390.1	Phosphoinositide phosphatase SAC1	_IGS(ph)GSDNLSSTMHR_	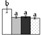
3110	KDP44118.1	Phosphoinositide phospholipase C 2	_RLS(ph)LSEPQLEK_	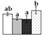
3180	KDP44771.1	Phosphoinositide phospholipase C 2-like	_GAS(ph)DEEAWGKEVSDLK_	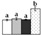
3364	KDP46231.1	phosphatidylinositol 4-kinase alpha 1	_LSGVGAAES(ph)K_	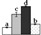
3551	KDP24987.1	Diacylglycerol kinase 5-like	_KFGAAPT(ph)FR_	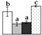
3975	KDP41761.1	Phosphatidylinositol phosphatidylcholine transfer protein SFH8	_LT(ph)PVREESK_	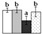
**Protein misfolding and degradation**				
126	KDP20698.1	Plant UBX domain-containing protein 4	_TLS(ph)DLNRR_	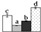
239	KDP21542.1	NPL4-like protein 1	_TIAGPAIHPAGS(ph)FGR_	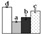
1207	KDP29157.1	Cell division cycle protein 48 homolog	_DFS(ph)TAILER_	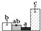
1583	KDP32077.1	Peptidyl-prolyl cis-trans isomerase cyp65	_S(ph)FTSTSFDPVTK_	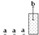
1216	KDP29225.1	E3 ubiquitin-protein ligase UPL7	_DLS(ph)LDFTVTEESFGKR_	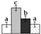
1276	KDP29647.1	E3 ubiquitin-protein ligase UPL3	_S(ph)SVNIGDAAR_	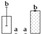
2820	KDP41504.1	E3 ubiquitin-protein ligase UPL2-like	_ANLGNVNAGS(ph)VHGK_	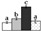
2907	KDP42227.1	E3 ubiquitin-protein ligase RING1	_NAGDRS(ph)PFNPVIVLR_	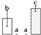
1279	KDP29680.1	Ubiquitin-conjugating enzyme E2 23	_LSPAAVSTS(ph)DSESAGELK_	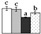
3548	KDP36146.1	ubiquitin-conjugating enzyme E2 variant 1D	_T(ph)LGSGGSSVVVPR_	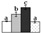
1272	KDP29632.1	26s proteasome non-atpase regulatory subunit 4 homolog	_VEEPSSTS(ph)QDATVVEK_	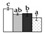
1742	KDP33301.1	26s protease regulatory subunit 6b homolog	_PVLEPLPS(ph)IPK_	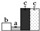
**14-3-3 Related**				
655	KDP25197.1	Serine threonine-protein kinase BLUS1	_ASANSLS(ph)APIK_	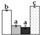
657	KDP25197.1	Serine threonine-protein kinase BLUS1	_KLPS(ph)FSGPLM(ox)LPNR_	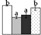
1724	KDP32938.1	14-3-3-like protein a	_MS(ph)PTETSR_	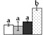
2765	KDP40925.1	14-3-3 protein 6	_(ac)AAGS(ph)PREDNVYMAK_	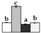
3118	KDP44148.1	Phototropin-1	_RNS(ph)ENVPSNR_	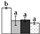
**HSPs**				
1375	KDP30625.1	Chaperonin 60 subunit beta chloroplastic	_LAS(ph)KVDAIK_	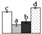
1658	KDP32613.1	Heat shock cognate protein 80	_EVSHEWS(ph)LVNK_	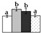
2898	KDP42168.1	kDa class I heat shock	_(ac)ALLPSFFGNS(ph)R_	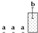
3205	KDP44981.1	Heat shock cognate 70 kDa protein 2-like	_RFS(ph)DASVQSDIK_	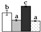
3206	KDP44981.1	Heat shock cognate 70 kDa protein 2-like	_FSDASVQS(ph)DIK_	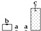
3984	KDP42448.1	Stromal 70 kDa heat shock-related chloroplastic	_LKT(ph)PVENSLR_	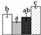
**Others**				
1743	KDP33317.1	Mitochondrial import receptor subunit TOM9-2	_TVS(ph)ESAVLNTAK_	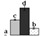
1984	KDP34991.1	ATP synthase subunit mitochondrial	_GQNVLNTGS(ph)PITVPVGR_	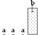
3239	KDP45196.1	Acyl-coenzyme a thioesterase mitochondrial	_(ac)MDFNSPS(ph)PR_	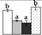
3307	KDP45748.1	Malonyl-acyl carrier protein mitochondrial	_LEAALAATAIKS(ph)PR_	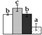
385	KDP22798.1	Regulatory-associated protein of TOR 1	_PGEPTTSS(ph)PTTSLAGLAR_	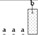
415	KDP23116.1	Autophagy-related protein 13	_GAPFTVNQPFGGS(ph)PPAYR_	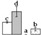

* The four columns from left to right were corresponding to C0 h, C6 h, C24 h and R24 h. The different lowercase letters labeled above columns indicate significant changes according to one-way ANOVA (*p* < 0.05).

## Supplementary Materials

Supplementary materials can be found at http://www.mdpi.com/1422-0067/20/1/208/s1.

## Abbreviations

GO	Gene Ontology
PS	Photosystem
ABA	Abscisic acid
SnRK2	Sucrose non-fermenting 1-related protein kinase 2
ABI5	ABA Insensitive 5
HOS1	High expression of osmotically responsive gene 1
CBF	C-repeat binding factor
ICE1	Inducer of CBF expression 1
LC-MS	Liquid chromatograph-mass spectrometer
CDK	Cyclin-dependent kinase
MAPK	Mitogen-activated protein kinase
CDPK	Calmodulin-dependent protein kinase
KEG	RING-type E3 ligase KEEP ON GOING
KOG	Eukaryotic orthologous groups
ABI5	ABA-insensitive 5 protein
EIN	Ethylene insensitive protein
MKP	Mitogen-activated protein kinase phosphatase
EDR	Enhanced disease resistance
PLC	Phosphoinositide phospholipase C
PI(4,5)P2	Phosphatidylinositol 4,5-bisphosphate
DAG	Diacylglycerol
PI4K	phosphatidylinositol 4-kinase
DAGK	Diacylglycerol kinase 5
PI4P	Phosphatidylinositol 4-phosphate
IP3	Inositol 1,4,5-trisphosphate
APX	Cytosolic ascorbate peroxidase
PHOT	Phototropin
CPN60B2	Chaperonin 60 subunit beta 2, chloroplastic
CHSP70	Stromal 70 kDa heat shock-related chloroplastic
Hsp90	Heat shock cognate protein 80
sHSP	17.3 kDa class I heat shock protein
PPI cyp65	Peptidyl-prolyl cis-trans isomerase cyp65
ER	Endoplasmic Reticulum
CHUP1	Chloroplast unusual positioning1
PMI	Plastid movement impaired

## References

[1] Thakur P., Kumar S., Malik J.A., Berger J.D., Nayyar H. (2010). Cold stress effects on reproductive development in grain crops: An overview. Environ. Exp. Bot..

[2] Levitt J. (1980). Chilling, Freezing and High Temperature Stresses. Responses of Plants to Environmental Stress.

[3] Dong C.H., Hu X., Tang W., Zheng X., Kim Y.S., Lee B.H., Zhu J.K. (2006). A putative Arabidopsis nucleoporin, AtNUP160, is critical for RNA export and required for plant tolerance to cold stress. Mol. Cell. Biol..

[4] Zhu J., Dong C.H., Zhu J.K. (2007). Interplay between cold-responsive gene regulation, metabolism and RNA processing during plant cold acclimation. Curr. Opin. Plant. Biol..

[5] Thomashow M.F. (1999). Plant Cold Acclimation: Freezing tolerance genes and regulatory mechanisms. Annu. Rev. Plant Physiol. Plant Mol. Biol..

[6] Huang W., Zhang S.B., Cao K.F. (2010). The different effects of chilling stress under moderate light intensity on photosystem II compared with photosystem I and subsequent recovery in tropical tree species. Photosynth. Res..

[7] Kratsch H.A., Wise R.R. (2000). The ultrastructure of chilling stress. Plant Cell Environ..

[8] Bascunan-Godoy L., Sanhueza C., Cuba M., Zuniga G.E., Corcuera L.J., Bravo L.A. (2012). Cold-acclimation limits low temperature induced photoinhibition by promoting a higher photochemical quantum yield and a more effective PSII restoration in darkness in the Antarctic rather than the Andean ecotype of Colobanthus quitensis Kunt Bartl (Cariophyllaceae). BMC Plant Biol..

[9] Shinozaki K., Yamaguchi-Shinozaki K. (1996). Molecular responses to drought and cold stress. Curr. Opin. Biotechnol..

[10] Ishitani M., Xiong L., Stevenson B., Zhu J.K. (1997). Genetic analysis of osmotic and cold stress signal transduction in Arabidopsis: Interactions and convergence of abscisic acid-dependent and abscisic acid-independent pathways. Plant Cell.

[11] Knight M.R., Campbell A.K., Smith S.M., Trewavas A.J. (1991). Transgenic plant aequorin reports the effects of touch and cold-shock and elicitors on cytoplasmic calcium. Nature.

[12] Knight H., Trewavas A.J., Knight M.R. (1996). Cold calcium signaling in Arabidopsis involves two cellular pools and a change in calcium signature after acclimation. Plant Cell.

[13] Monroy A.F., Dhindsa R.S. (1995). Low-temperature signal transduction: Induction of cold acclimation-specific genes of alfalfa by calcium at 25 °C. Plant Cell.

[14] Yamaguchi-Shinozaki K., Shinozaki K. (2006). Transcriptional regulatory networks in cellular responses and tolerance to dehydration and cold stresses. Annu. Rev. Plant Biol..

[15] Kobayashi Y., Yamamoto S., Minami H., Kagaya Y., Hattori T. (2004). Differential activation of the rice sucrose nonfermenting1-related protein kinase2 family by hyperosmotic stress and abscisic acid. Plant Cell.

[16] Kagaya Y., Hobo T., Murata M., Ban A., Hattori T. (2002). Abscisic acid-induced transcription is mediated by phosphorylation of an abscisic acid response element binding factor, TRAB1. Plant Cell.

[17] Hobo T., Kowyama Y., Hattori T. (1999). A bZIP factor, TRAB1, interacts with VP1 and mediates abscisic acid-induced transcription. Proc. Natl. Acad. Sci. USA.

[18] Finkelstein R.R., Lynch T.J. (2000). The Arabidopsis abscisic acid response gene ABI5 encodes a basic leucine zipper transcription factor. Plant Cell.

[19] Miura K., Lee J., Jin J.B., Yoo C.Y., Miura T., Hasegawa P.M. (2009). Sumoylation of ABI5 by the Arabidopsis SUMO E3 ligase SIZ1 negatively regulates abscisic acid signaling. Proc. Natl. Acad. Sci. USA.

[20] Ishitani M., Xiong L., Lee H., Stevenson B., Zhu J.K. (1998). HOS1, a genetic locus involved in cold-responsive gene expression in arabidopsis. Plant Cell.

[21] Dong C.H., Agarwal M., Zhang Y., Xie Q., Zhu J.K. (2006). The negative regulator of plant cold responses, HOS1, is a RING E3 ligase that mediates the ubiquitination and degradation of ICE1. Proc. Natl. Acad. Sci. USA.

[22] Kline-Jonakin K.G., Barrett-Wilt G.A., Sussman M.R. (2011). Quantitative plant phosphoproteomics. Curr. Opin. Plant Biol..

[23] Tichy A., Salovska B., Rehulka P., Klimentova J., Vavrova J., Stulik J., Hernychova L. (2011). Phosphoproteomics: Searching for a needle in a haystack. J. Proteomics.

[24] Kersten B., Agrawal G.K., Iwahashi H., Rakwal R. (2006). Plant phosphoproteomics: A long road ahead. Proteomics.

[25] Kersten B., Agrawal G.K., Durek P., Neigenfind J., Schulze W., Walther D., Rakwal R. (2009). Plant phosphoproteomics: An update. Proteomics.

[26] Rampitsch C., Bykova N.V. (2012). The beginnings of crop phosphoproteomics: Exploring early warning systems of stress. Front Plant Sci..

[27] Pi Z., Zhao M.L., Peng X.J., Shen S.H. (2017). Phosphoproteomic analysis of paper mulberry reveals phosphorylation functions in chilling tolerance. J. Proteome Res..

[28] Zhang M., Ma C.Y., Lv D.W., Zhen S.M., Li X.H., Yan Y.M. (2014). Comparative Phosphoproteome Analysis of the Developing Grains in Bread Wheat (*Triticum aestivum* L.) under Well-Watered and Water-Deficit Conditions. J. Proteome Res..

[29] Lv D.W., Subburaj S., Cao M., Yan X., Li X.H., Appels R., Sun D.F., Ma W.J., Yan Y.M. (2014). Proteome and phosphoproteome characterization reveals new response and defense mechanisms of *Brachypodium distachyon* leaves under salt stress. Mol. Cell. Proteom..

[30] Umezawa T., Sugiyama N., Takahashi F., Anderson J.C., Ishihama Y., Peck S.C., Shinozaki K. (2013). Genetics and phosphoproteomics reveal a protein phosphorylation network in the abscisic acid signaling pathway in *Arabidopsis thaliana*. Sci. Signal..

[31] Heller J. (1996). Physic Nut. Jatropha curcas L. Promoting the Conservation and Use of Underutilized and Neglected Crops.

[32] Fairless D. (2007). Biofuel: The little shrub that could—Maybe. Nature.

[33] Sato S., Hirakawa H., Isobe S., Fukai E., Watanabe A., Kato M., Kawashima K., Minami C., Muraki A., Nakazaki N. (2010). Sequence analysis of the genome of an oil-bearing tree, *Jatropha curcas* L.. DNA Res..

[34] Carvalho C.R., Clarindo W.R., Praça M.M., Araújo F.S., Carels N. (2008). Genome size, base composition and karyotype of *Jatropha curcas* L., an important biofuel plant. Plant Sci..

[35] Jiang H., Wu P., Zhang S., Song C., Chen Y., Li M., Jia Y., Fang X., Chen F., Wu G. (2012). Global analysis of gene expression profiles in developing physic nut (*Jatropha curcas* L.) seeds. PLoS ONE.

[36] Zhang L., Zhang C., Wu P., Chen Y., Li M., Jiang H., Wu G. (2014). Global analysis of gene expression profiles in physic nut (*Jatropha curcas* L.) seedlings exposed to salt stress. PLoS ONE.

[37] Liu H., Liu Y.J., Yang M.F., Shen S.H. (2009). A comparative analysis of embryo and endosperm proteome from seeds of *Jatropha curcas*. J. Integr. Plant Biol..

[38] Liu H., Wang C., Chen F., Shen S. (2015). Proteomic analysis of oil bodies in mature *Jatropha curcas* seeds with different lipid content. J. Proteom..

[39] Liu H., Wang C., Komatsu S., He M., Liu G., Shen S. (2013). Proteomic analysis of the seed development in *Jatropha curcas*: From carbon flux to the lipid accumulation. J. Proteom..

[40] Liu H., Yang Z., Yang M., Shen S. (2011). The differential proteome of endosperm and embryo from mature seed of *Jatropha curcas*. Plant Sci..

[41] Yang M.F., Liu Y.J., Liu Y., Chen H., Chen F., Shen S.H. (2009). Proteomic analysis of oil mobilization in seed germination and postgermination development of *Jatropha curcas*. J. Proteome Res..

[42] Wang H., Zou Z., Wang S., Gong M. (2013). Global analysis of transcriptome responses and gene expression profiles to cold stress of *Jatropha curcas* L.. PLoS ONE.

[43] Liang Y., Chen H., Tang M.J., Yang P.F., Shen S.H. (2007). Responses of *Jatropha curcas* seedlings to cold stress: Photosynthesis-related proteins and chlorophyll fluorescence characteristics. Physiol. Plant.

[44] Yan S.P., Zhang Q.Y., Tang Z.C., Su W.A., Sun W.N. (2006). Comparative proteomic analysis provides new insights into chilling stress responses in rice. Mol. Cell. Proteom..

[45] Yang Q.S., Wu J.H., Li C.Y., Wei Y.R., Sheng O., Hu C.H., Kuang R.B., Huang Y.H., Peng X.X., McCardle J.A. (2012). Quantitative proteomic analysis reveals that antioxidation mechanisms contribute to cold tolerance in plantain (*Musa paradisiaca* L.; ABB Group) seedlings. Mol. Cell. Proteom..

[46] Vizcaino J.A., Cote R.G., Csordas A., Dianes J.A., Fabregat A., Foster J.M., Griss J., Alpi E., Birim M., Contell J. (2013). The Proteomics Identifications (PRIDE) database and associated tools: Status in 2013. Nucleic Acids Res..

[47] Yao Q., Bollinger C., Gao J., Xu D., Thelen J.J. (2012). P^3^DB: An integrated database for plant protein phosphorylation. Front Plant Sci..

[48] Heazlewood J.L., Durek P., Hummel J., Selbig J., Weckwerth W., Walther D., Schulze W.X. (2008). PhosPhAt: A database of phosphorylation sites in *Arabidopsis thaliana* and a plant-specific phosphorylation site predictor. Nucleic Acids Res..

[49] Schwartz D., Gygi S.P. (2005). An iterative statistical approach to the identification of protein phosphorylation motifs from large-scale data sets. Nat. Biotechnol..

[50] Amanchy R., Periaswamy B., Mathivanan S., Reddy R., Tattikota S.G., Pandey A. (2007). A curated compendium of phosphorylation motifs. Nat. Biotechnol..

[51] Prasad T.S.K., Goel R., Kandasamy K., Keerthikumar S., Kumar S., Mathivanan S., Telikicherla D., Raju R., Shafreen B., Venugopal A. (2009). Human protein reference database-2009 update. Nucleic Acids Res..

[52] Villen J., Beausoleil S.A., Gerber S.A., Gygi S.P. (2007). Large-scale phosphorylation analysis of mouse liver. Proc. Natl. Acad. Sci. USA.

[53] Ku N.O., Liao J., Omary M.B. (1998). Phosphorylation of human keratin 18 serine 33 regulates binding to 14-3-3 proteins. EMBO J..

[54] Zhang S.H., Kobayashi R., Graves P.R., PiwnicaWorms H., Tonks N.K. (1997). Serine phosphorylation-dependent association of the band 4.1-related protein-tyrosine phosphatase PTPH1 with 14-3-3 beta protein. J. Biol. Chem..

[55] Vlad F., Turk B.E., Peynot P., Leung J., Merlot S. (2008). A versatile strategy to define the phosphorylation preferences of plant protein kinases and screen for putative substrates. Plant J..

[56] Boudsocq M., Barbier-Brygoo H., Lauriere C. (2004). Identification of nine sucrose nonfermenting 1-related protein kinases 2 activated by hyperosmotic and saline stresses in *Arabidopsis thaliana*. J. Biol. Chem..

[57] Mao X., Zhang H., Tian S., Chang X., Jing R. (2010). TaSnRK2.4, an SNF1-type serine/threonine protein kinase of wheat (*Triticum aestivum* L.), confers enhanced multistress tolerance in Arabidopsis. J. Exp. Bot..

[58] Hrabak E.M. (2003). The Arabidopsis CDPK-SnRK superfamily of protein kinases. Plant Physiol..

[59] Baena-Gonzalez E., Rolland F., Thevelein J.M., Sheen J. (2007). A central integrator of transcription networks in plant stress and energy signalling. Nature.

[60] Hardie D.G., Carling D., Carlson M. (1998). The AMP-activated/SNF1 protein kinase subfamily: Metabolic sensors of the eukaryotic cell?. Annu. Rev. Biochem..

[61] Qin F., Shinozaki K., Yamaguchi-Shinozaki K. (2011). Achievements and challenges in understanding plant abiotic stress responses and tolerance. Plant Cell Physiol..

[62] Liu H., Stone S.L. (2010). Abscisic acid increases Arabidopsis ABI5 transcription factor levels by promoting KEG E3 ligase self-ubiquitination and proteasomal degradation. Plant Cell.

[63] Kobayashi Y., Murata M., Minami H., Yamamoto S., Kagaya Y., Hobo T., Yamamoto A., Hattori T. (2005). Abscisic acid-activated SnRK2 protein kinases function in the gene-regulation pathway of ABA signal transduction by phosphorylating ABA response element-binding factors. Plant J..

[64] Sanders D., Brownlee C., Harper J.F. (1999). Communicating with calcium. Plant Cell.

[65] Li W.G., Komatsu S. (2000). Cold stress induced calcium-dependent protein kinase(s) in rice (*Oryza sativa* L.) seedling stem tissues. Theor. Appl. Genet..

[66] Khan M., Takasaki H., Komatsu S. (2005). Comprehensive phosphoproteome analysis in rice and identification of phosphoproteins responsive to different hormones/stresses. J. Proteome Res..

[67] Hua J., Meyerowitz E.M. (1998). Ethylene responses are negatively regulated by a receptor gene family in *Arabidopsis thaliana*. Cell.

[68] Zheng Y., Zhu Z. (2016). Relaying the ethylene signal: New roles for EIN2. Trends Plant Sci..

[69] Solanke A.U., Sharma A.K. (2008). Signal transduction during cold stress in plants. Physiol. Mol. Biol. Plants.

[70] Yadav S.K. (2010). Cold stress tolerance mechanisms in plants. A review. Agron. Sustain. Dev..

[71] Chinnusamy V., Zhu J.K., Sunkar R. (2010). Gene regulation during cold stress acclimation in plants. Methods Mol. Biol..

[72] Jonak C., Kiegerl S., Ligterink W., Barker P.J., Huskisson N.S., Hirt H. (1996). Stress signaling in plants: A mitogen-activated protein kinase pathway is activated by cold and drought. Proc. Natl. Acad. Sci. USA.

[73] Keyse S.M. (2000). Protein phosphatases and the regulation of mitogen-activated protein kinase signalling. Curr. Opin. Cell Biol..

[74] Boss W.F., Im Y.J. (2012). Phosphoinositide signaling. Annu. Rev. Plant Biol..

[75] Kanehara K., Yu C.Y., Cho Y., Cheong W.F., Torta F., Shui G., Wenk M.R., Nakamura Y. (2015). Arabidopsis AtPLC2 is a primary phosphoinositide-specific Phospholipase C in phosphoinositide metabolism and the endoplasmic reticulum stress response. PLoS Genet..

[76] Kim D.H., Eu Y.J., Yoo C.M., Kim Y.W., Pih K.T., Jin J.B., Kim S.J., Stenmark H., Hwang I. (2001). Trafficking of phosphatidylinositol 3-phosphate from the trans-Golgi network to the lumen of the central vacuole in plant cells. Plant Cell.

[77] Hirayama T., Mitsukawa N., Shibata D., Shinozaki K. (1997). *AtPLC2*, a gene encoding phosphoinositide-specific phospholipase C, is constitutively expressed in vegetative and floral tissues in *Arabidopsis thaliana*. Plant Mol. Biol..

[78] Chen Y., Hoehenwarter W., Weckwerth W. (2010). Comparative analysis of phytohormone-responsive phosphoproteins in Arabidopsis thaliana using TiO_2_-phosphopeptide enrichment and mass accuracy precursor alignment. Plant J..

[79] Singh A., Bhatnagar N., Pandey A., Pandey G.K. (2015). Plant phospholipase C family: Regulation and functional role in lipid signaling. Cell Calcium.

[80] Delage E., Ruelland E., Guillas I., Zachowski A., Puyaubert J. (2012). Arabidopsis type-III phosphatidylinositol 4-kinases beta1 and beta2 are upstream of the phospholipase C pathway triggered by cold exposure. Plant Cell Physiol..

[81] Delage E., Puyaubert J., Zachowski A., Ruelland E. (2013). Signal transduction pathways involving phosphatidylinositol 4-phosphate and phosphatidylinositol 4,5-bisphosphate: Convergences and divergences among eukaryotic kingdoms. Prog. Lipid Res..

[82] Aroca R., Amodeo G., Fernandez-Illescas S., Herman E.M., Chaumont F., Chrispeels M.J. (2005). The role of aquaporins and membrane damage in chilling and hydrogen peroxide induced changes in the hydraulic conductance of maize roots. Plant Physiol..

[83] Chrispeels M.J., Agre P. (1994). Aquaporins: Water channel proteins of plant and animal cells. Trends Biochem. Sci..

[84] Raven E.L. (2003). Understanding functional diversity and substrate specificity in haem peroxidases: What can we learn from ascorbate peroxidase?. Nat. Prod. Rep..

[85] Takemiya A., Sugiyama N., Fujimoto H., Tsutsumi T., Yamauchi S., Hiyama A., Tada Y., Christie J.M., Shimazaki K.I. (2013). Phosphorylation of BLUS1 kinase by phototropins is a primary step in stomatal opening. Nat. Commun..

[86] Li C.L., Wang M., Wu X.M., Chen D.H., Lv H.J., Shen J.L., Qiao Z., Zhang W. (2016). THI1, a thiamine thiazole synthase, interacts with Ca^2+^-dependent protein kinase CPK33 and modulates the S-type anion channels and stomatal closure in Arabidopsis. Plant Physiol..

[87] Roberts M.R., Salinas J., Collinge D.B. (2002). 14-3-3 proteins and the response to abiotic and biotic stress. Plant Mol. Biol..

[88] Viitanen P.V., Schmidt M., Buchner J., Suzuki T., Vierling E., Dickson R., Lorimer G.H., Gatenby A., Soll J. (1995). Functional characterization of the higher plant chloroplast chaperonins. J. Biol. Chem..

[89] Gething M.J., Sambrook J. (1992). Protein folding in the cell. Nature.

[90] Salvucci M.E. (2008). Association of RuBisCo activase with chaperonin-60beta: A possible mechanism for protecting photosynthesis during heat stress. J. Exp. Bot..

[91] Deng Y., Srivastava R., Howell S.H. (2013). Endoplasmic reticulum (ER) stress response and its physiological roles in plants. Int. J. Mol. Sci..

[92] Sonoike K. (1998). Various aspects of inhibition of photosynthesis under light/chilling stress: “Photoinhibition at chilling temperatures” versus “chilling damage in the light”. J. Plant Res..

[93] Powles S.B. (1984). Photoinhibition of photosynthesis induced by visible light. Annu. Rev. Plant Physiol..

[94] Salonen M., Aro E.M., Rintamaki E. (1998). Reversible phosphorylation and turnover of the D1 protein under various redox states of Photosystem II induced by low temperature photoinhibition. Photosynth. Res..

[95] Nishiyama Y., Allakhverdiev S.I., Yamamoto H., Hayashi H., Murata N. (2004). Singlet oxygen inhibits the repair of photosystem II by suppressing the translation elongation of the D1 protein in synechocystis sp. PCC 6803. Biochemistry.

[96] Hong J.H., Chang C.X., Moon B.Y., Lee C.H. (2003). Recovery from low-temperature photoinhibition is related to dephosphorylation of phosphorylated CP29 rather than zeaxanthin epoxidation in rice leaves. J. Plant Biol..

[97] Davis P.A., Hangarter R.P. (2012). Chloroplast movement provides photoprotection to plants by redistributing PSII damage within leaves. Photosynth. Res..

[98] Sakai T., Kagawa T., Kasahara M., Swartz T.E., Christie J.M., Briggs W.R., Wada M., Okada K. (2001). Arabidopsis nph1 and npl1: Blue light receptors that mediate both phototropism and chloroplast relocation. Proc. Natl. Acad. Sci. USA.

[99] Labuz J., Hermanowicz P., Gabrys H. (2015). The impact of temperature on blue light induced chloroplast movements in *Arabidopsis thaliana*. Plant Sci..

[100] Banas A.K., Aggarwal C., Labuz J., Sztatelman O., Gabrys H. (2012). Blue light signalling in chloroplast movements. J. Exp. Bot..

[101] Christie J.M. (2007). Phototropin blue-light receptors. Annu. Rev. Plant Biol..

[102] Shen S., Jing Y., Kuang T. (2003). Proteomics approach to identify wound-response related proteins from rice leaf sheath. Proteomics.

[103] Ramagli L.S. (1999). Quantifying protein in 2-D PAGE solubilization buffers. Methods Mol. Biol..

[104] Olsen J.V., Blagoev B., Gnad F., Macek B., Kumar C., Mortensen P., Mann M. (2006). Global, in vivo and site-specific phosphorylation dynamics in signaling networks. Cell.

