# Supplementary material for: Global Phosphoproteomic Analysis Reveals the Defense and Response Mechanisms of Jatropha Curcas Seedling under Chilling Stress
Source: Int J Mol Sci. 2019 Jan 8;20(1):208. doi: 10.3390/ijms20010208 (PMC6337099; doi:10.3390/ijms20010208)
Supplement: Supplementary file 1 [file ijms-20-00208-s001.zip › Supplemental files/Figure S1-S6.pdf]

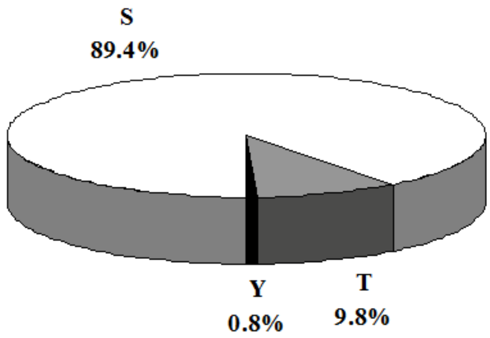

Figure S1. The proportions of phosphorylation sites of Ser, Thr and Tyr from 3101 identified phosphopeptides in *J. curcas* seedling under chilling treatment and recovery.

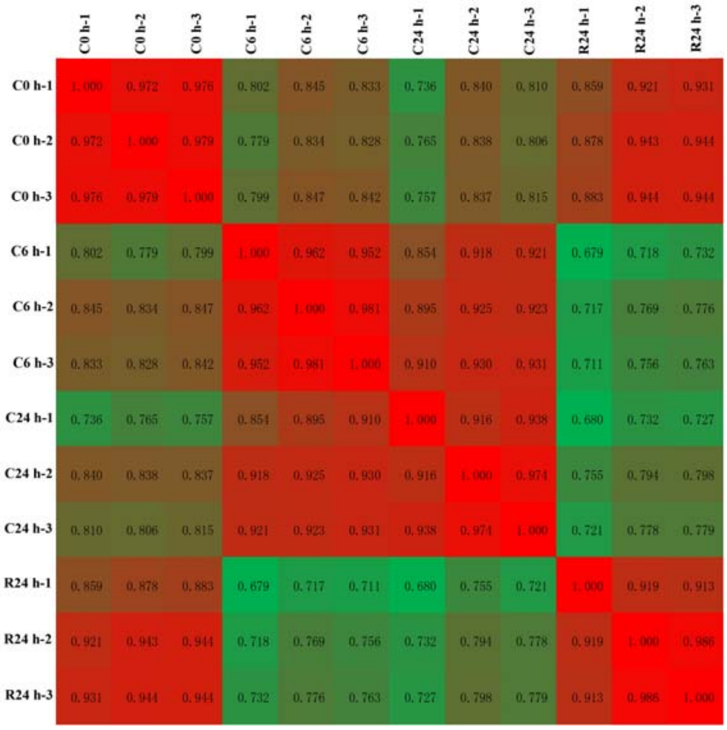

Figure S2. The Pearson correlation analysis of the four experimental samples (C0 h, C6 h, C24 h and R24 h). The results showed that the four samples were individually clustered confidently with their replicates.

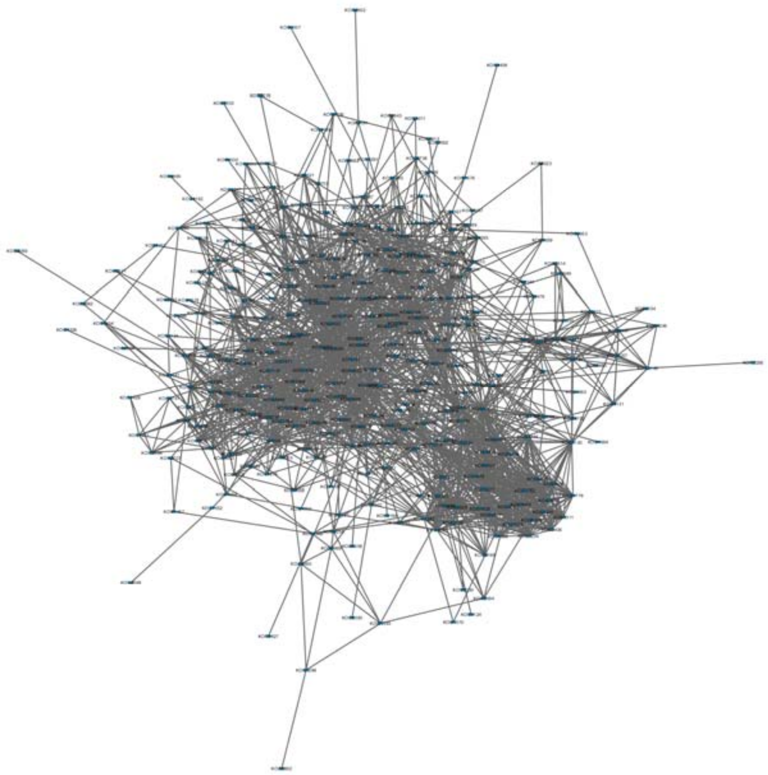

Figure S3. Protein-protein interaction (PPI) network of 514 KOGs representing 610 phosphoproteins. This network contained 319 nodes and 1924 edges, and the confidence score was set at the highest level (≥0.900).

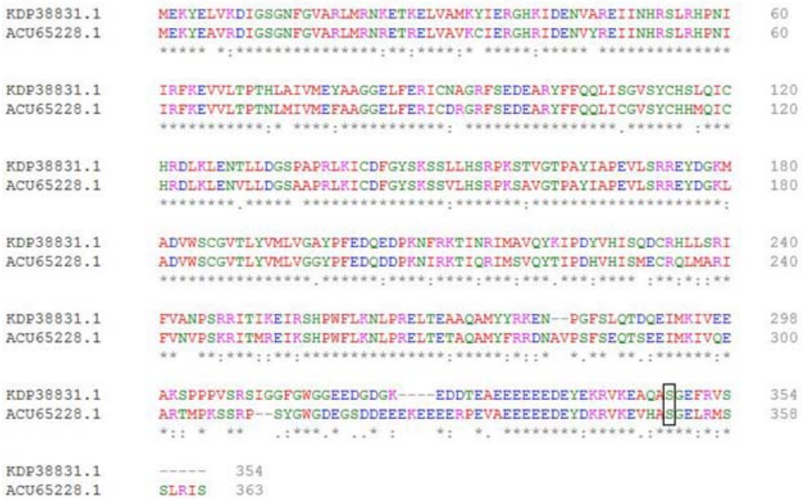

Figure S4. Sequence alignment of JcSnRK2a (KDP38831.1) and TaSnRK2.4 (ACU65228.1). The numbers on the right indicate the amino acid position. Identical amino acid residues are labeled with same color. The Ser 348 was phosphorylated in *J. curcas* seedling under chilling stress.

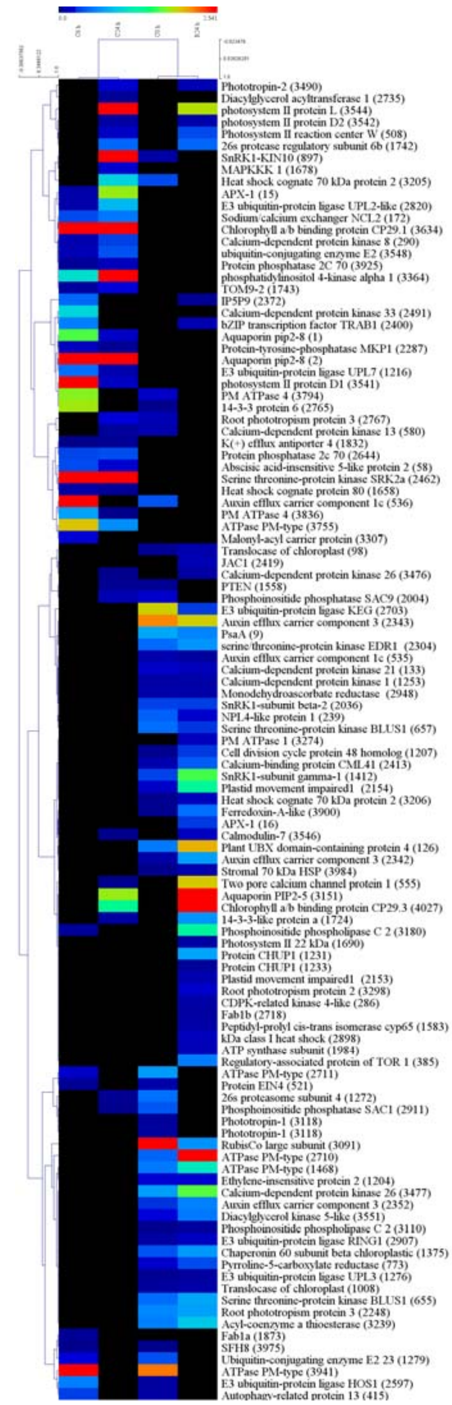

Figure S5. Cluster analysis of abundance profiles of 111 phosphoproteins with significantly change at phosphorylation level related to the response- and defense- network of *J. curcas* seedling under chilling treatment. This heat map was plotted by employing MultiExperiment Viewer 4.9.0. The value of phosphoprotein was average and "0" value was replaced by a minimal value, 10^{-9} to indicate the biological significance. The id number in bracket is corresponding to the description in Table 1.

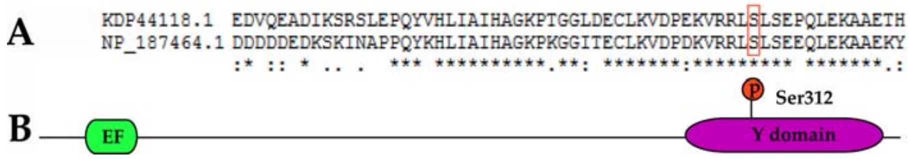

Figure S6. Sequence alignment and structural composition of PLC2 in *J. curcas* seedling. (A) JcPLC2 sequence alignment with AtPLC2. (B) Predicted function domain distribution of JcPLC2.
